# Organoid Technology: A Reliable Developmental Biology Tool for Organ-Specific Nanotoxicity Evaluation

**DOI:** 10.3389/fcell.2021.696668

**Published:** 2021-09-23

**Authors:** Minakshi Prasad, Rajesh Kumar, Lukumoni Buragohain, Ankur Kumari, Mayukh Ghosh

**Affiliations:** ^1^Department of Animal Biotechnology, Lala Lajpat Rai University of Veterinary and Animal Sciences, Hisar, India; ^2^Department of Veterinary Physiology and Biochemistry, Lala Lajpat Rai University of Veterinary and Animal Sciences, Hisar, India; ^3^Department of Animal Biotechnology, College of Veterinary Science, Assam Agricultural University, Guwahati, India; ^4^Department of Zoology, CBLU, Haryana, India; ^5^Department of Veterinary Physiology and Biochemistry, RGSC, Banaras Hindu University, Varanasi, India

**Keywords:** nanotoxicity, nanoparticle, genotoxicity, organoids, 2D monocultures, microfluidics, spheroids

## Abstract

Engineered nanomaterials are bestowed with certain inherent physicochemical properties unlike their parent materials, rendering them suitable for the multifaceted needs of state-of-the-art biomedical, and pharmaceutical applications. The log-phase development of nano-science along with improved “bench to beside” conversion carries an enhanced probability of human exposure with numerous nanoparticles. Thus, toxicity assessment of these novel nanoscale materials holds a key to ensuring the safety aspects or else the global biome will certainly face a debacle. The toxicity may span from health hazards due to direct exposure to indirect means through food chain contamination or environmental pollution, even causing genotoxicity. Multiple ways of nanotoxicity evaluation include several *in vitro* and *in vivo* methods, with *in vitro* methods occupying the bulk of the “experimental space.” The underlying reason may be multiple, but ethical constraints in *in vivo* animal experiments are a significant one. Two-dimensional (2D) monoculture is undoubtedly the most exploited *in vitro* method providing advantages in terms of cost-effectiveness, high throughput, and reproducibility. However, it often fails to mimic a tissue or organ which possesses a defined three-dimensional structure (3D) along with intercellular communication machinery. Instead, microtissues such as spheroids or organoids having a precise 3D architecture and proximate *in vivo* tissue-like behavior can provide a more realistic evaluation than 2D monocultures. Recent developments in microfluidics and bioreactor-based organoid synthesis have eased the difficulties to prosper nano-toxicological analysis in organoid models surpassing the obstacle of ethical issues. The present review will enlighten applications of organoids in nanotoxicological evaluation, their advantages, and prospects toward securing commonplace nano-interventions.

## Introduction

Nanotechnology empowered by engineered nanomaterials has almost left no stone untouched in the scientific arena of the current century. Nanomaterials are ascribed with a nanoscale range external/internal structure at least in one dimension, which adorns them with distinct physicochemical properties unlike their bulk equivalents (Taniguchi, 1974; Laurent et al., 2010; Drasler et al., 2017). Further, the nanomaterial repository is prospering apace propelled by novel functionalization methods and derived nano-entities with newer attributes (Yan et al., 2020; Ji et al., 2021). These innovative materials with diverse attributes such as small size facilitating cellular uptake, high surface-to-volume ratio promoting ample surface functionalization and precise molecular interaction, and unique light scattering for molecular imaging have paved the way toward widespread nano-intervention in numerous state-of-the-art technological developments (Murphy et al., 2015; Yaqoob et al., 2020). The applications spanned over material science to engineering, in energy harvesting to agriculture including biomedical and pharmaceutical utility (Jariwala et al., 2013; Chattopadhyay et al., 2017; Minakshi et al., 2020a). Nanotechnology has achieved extensive penetration in almost every branch of healthcare and biomedical science ranging from intelligent vaccine formulations, state-of-the-art diagnostics to advanced therapeutics, particularly as targeted drug delivery and sustained drug release systems, even in monitoring the disease progression and therapeutic outcome (Salata, 2004; Murthy, 2007; De Jong and Borm, 2008; Rizzo et al., 2013; Lombardo et al., 2019; Minakshi et al., 2019, 2020b, c; Tharkar et al., 2019; Chauhan et al., 2020).

**FIGURE 1:**
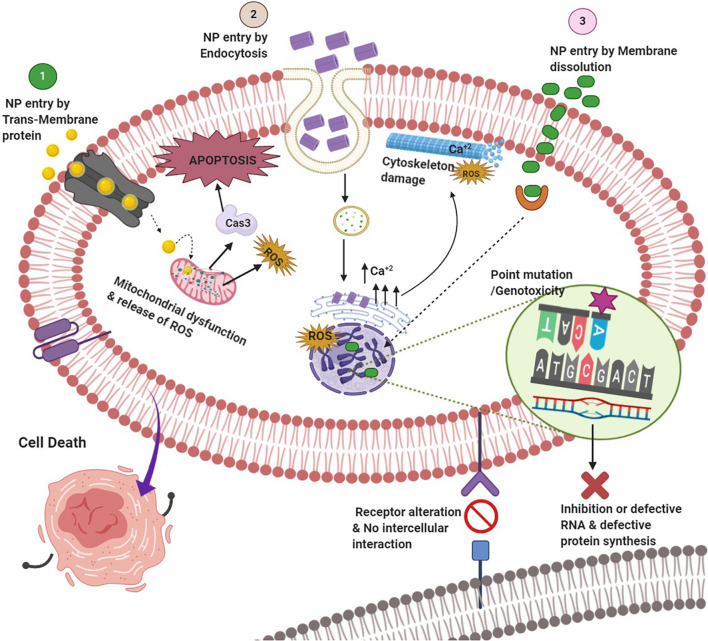
Potential routes of nanoparticle entry and molecular mechanisms of nanotoxicity.

Despite stringent regulations toward nano-intervention in biomedical applications, nano-science has achieved considerable improvement in “bench to beside” transition in recent times (Hua et al., 2018; Rosenblum et al., 2018). Evidently, the probability of human exposure with myriads of nano-formulations has augmented significantly (Yan et al., 2020). Short- as well as long-term toxicity assessment of these novel nano-formulations is of paramount importance to ensure the safety of the global biome (Gatoo et al., 2014; Garduño-Balderas et al., 2015; Ji et al., 2021). The source of these nanoparticles (NPs) may be natural such as volcanic eruptions and photo-oxidation or anthropogenic processes including increased use of nanomaterials and their waste or residue generation (Bystrzejewska-Piotrowska et al., 2009; Luo et al., 2016). A plethora of studies has depicted toxicities associated with NPs, which has attracted concern from various stakeholders (Bahadar et al., 2016; Crisponi et al., 2017; Buchman et al., 2019; Yang et al., 2020). The toxicity may be inflicted through either health hazards due to direct exposure or indirect toxicity by food chain contamination or environmental pollution. The usual mechanisms of nanotoxicity include but are not limited to cytotoxicity, genotoxicity, production of reactive oxygen species (ROS), oxidative stress and inflammation, modulation of cell signaling, apoptosis, and cancer (Figure 1; Rowinsky and Donehower, 1995; Joris et al., 2013; Fu et al., 2014; Alphandéry et al., 2015; Wang et al., 2015; Yang et al., 2020). Even nanotherapeutics approved by the US Food and Drug Administration (FDA) for clinical use are not entirely free from the potentially toxic effects (Table 1). Hence, effective and reliable methods for nanotoxicity evaluation at cellular or organ level is required to escape from the untoward effects of the emerging nanoformulations.

**TABLE 1:** Toxic effects of nanotherapeutics approved by FDA for clinical use.

Nanoparticle-based drugs	Application	Toxic/adverse effects observed
Abelcet (liposome)	Antifungal, cryptococcal meningitis in HIV	Anaphylaxis
Abraxane (albumin)	Metastatic breast cancer	Myelosuppression, sepsis, pneumonitis, and fetal harm
Adagen (polymeric NPs) Monomethoxypolyethylene glycol succinimidyl)	SCID, support therapy for bone marrow transplantation	Due to shortage of API
Adynovate (PEGylation)	Hemophilia A	Inhibitor formation and hypersensitivity reactions
AmBIsome (liposome)	Antifungal/protozoal infections	Elderly patients and hepatic impairment is known
Avinza (nanocrystal)	Sever pain	Addiction, fatal respiratory depression, and neonatal problem
Caelyx^®^ (liposome)	Opportunistic infections and coinfections, and HIV patient secondary infection	Dermal lesions primarily on the feet and legs
Cimzia^®^ or certolizumab pegol (BSA) (PEGylated)	Crohn’s disease, rheumatoid arthritis, psoriatic arthritis, ankylosing spondylitis, plaque psoriasis, and non-radiographic axial spondyloarthritis	Tuberculosis (TB), bacterial sepsis, and invasive fungal infections
Copaxone (polymer NPs)	Relapsing–remitting multiple sclerosis	Chest pain, lipoatrophy, and skin necrosis
Cremophor^®^ EL (PEG)	Cancer	Hypersensitivity reactions and cardiac arrest
Curosurf (liposome)	Respiratory distress syndrome	Acute changes in lung compliance
Cynviloq (PEG-PDLLA)	Metastatic breast cancer (MBC) and non-small cell lung cancer (NSCLC)	Data still not available
DaunoXome (Liposome)	HIV-related kaposi sarcoma	Cardiac function, severe myelosuppression, and hepatotoxicity
DepoCyt (liposomal cytarabine)	Lymphomatous meningitis	Cauda equina syndrome, visual disturbances, and arachnoiditis
DepoDur (liposomal)	Pain (postoperative analgesic)	Increased sensitivity in elderly, hepatic, and renal failure in compromised patient
Eligard [polylactide-co-glycolic acid (PLGA)]	Advanced prostate cancer	Irritation and erythema, ureteral obstruction, and/or spinal cord compression, increase testosterone
Estrasorb (micelles)	Vasomotor symptoms due to menopause	Endometrial cancer, cardiovascular disorders, breast cancer, and probable dementia
Feraheme (iron oxide NPs)	Iron deficiency anemia	Constipation, diarrhea, edema, hypotension, anaphylactic-like reactions, and hypotension
Feridex (iron oxide NP)	Advanced prostate cancer, magnetic resonance imaging contrast	Anaphylactic-like reactions and hypotension
GastroMARK (iron oxide nanoparticles)	Magnetic resonance imaging contrast	As made earlier not recommended for iron supplementation
Genexol-PM (poly(ethylene glycol)-block-poly(D,L-lactide) (PEG-PDLLA)	Metastatic breast cancer (MBC)	Data still not available
Krystexxa^®^ (PEGylated)	Gout	Gout flares and infusion-related reactions (IRs), serious allergic reactions
Macugen (polymeric NPs)	Neovascular AMD	Endophthalmitis
Mircera^®^ or Methoxy PEGepoetin	Anemia associated with chronic kidney disease	Myocardial infarction, stroke, venous thromboembolism, thrombosis of vascular access and tumor progression or recurrence, hypertension
Myocet and DaunoXome (liposomal)	Anticancer	Neutropenic fever, sepsis, stomatitis, alopecia, and bone marrow suppression
Oncaspar (PEGasparaginase)	Acute lymphoblastic leukemia	Pancreatitis, anaphylaxis, glucose intolerance, and coagulopathy
Plegridy^®^ (Biogene) (PEG)	Relapsing multiple sclerosis	Thrombotic microangiopathy
Tricor (nanocrystal)	Hyperlipidemia	Liver problems, breathing problems, abdominal pain, muscle problems, and nausea
Vitoss (nanocrystal)	Bone void filler	No data

**FIGURE 2:**
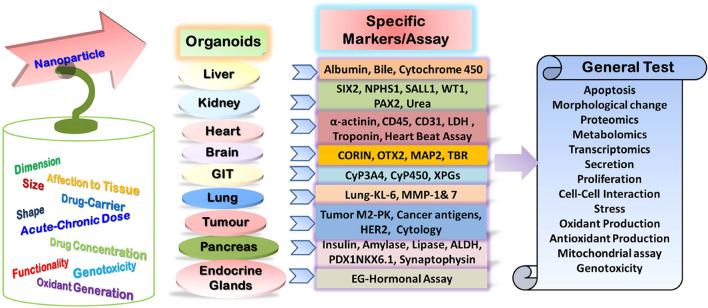
Potential specific and general tests for assessment of nanoparticle toxicity in the organoid model.

Several *in vitro* as well as *in vivo* methods provide the scope for nanotoxicity evaluation in a conventional way (Figure 2; Hillegass et al., 2010; Arora et al., 2012; Chuang et al., 2013; Astashkina et al., 2014; Kumar et al., 2017; Shinde et al., 2020). Cytotoxicity analyses along with observations regarding genotoxicity, inflammation, and oxidative stress in cell cultures are the most convenient tools for *in vitro* nanotoxicity assessment (Hillegass et al., 2010; Arora et al., 2012; Astashkina et al., 2014; Kumar et al., 2017). Determination of LD_50_, measuring biodistribution and clearance, morphological analyses of tissues or organs, and hemato-biochemical estimations in model organisms are the most common classical *in vivo* methods employed to serve the purpose (Jones and Grainger, 2009; Kumar et al., 2017). However, these methods provide toxicity signals much later, mostly after phenotypic impressions have been panned out, and often skip the early signs particularly in low-dose toxicity (Jones and Grainger, 2009; Kumar et al., 2017). Two-dimensional (2D) monoculture is arguably the most preferred *in vitro* nanotoxicity assessment modality. The modality possesses multiple advantages including the popularity of cell culture-based methods, prominence, cost-effectiveness, high throughput, reproducibility, and most importantly, being devoid of the ethical constraints associated with animal and human experiments (Evans et al., 2016; Huang et al., 2021). However, the lack of defined three-dimensional (3D) structures and cellular cross-talking networks pose limitations to this modality. Thus, it often fails to replicate *in vivo* tissue or organ microenvironment and behavior (Evans et al., 2016; Huang et al., 2021). Recent developments to customize micro-tissues such as spheroids or organoids can overcome the limitations to provide a more pragmatic evaluation. So, these models are progressively getting established as an efficient replica for disease modeling, drug testing and toxicity assessment, and regenerative and personalized medicine (Jensen and Teng, 2020; Rodriguez-Garcia et al., 2020; Velasco V. et al., 2020; Gunti et al., 2021). Organoids can provide additional benefits in terms of having a relatively long life than monolayer cultures and being devoid of ethical constraints and maintenance issues of laboratory animals. They also mimic the structural, functional, and genotypic properties of respective organs (Hartung and Daston, 2009; Kim et al., 2020). Further, the best advantage of using organoids over conventional *in vivo* lab animal experiments can be observed, as organoids can be developed directly from the targeted organism as inter-organism differences in drug metabolism can be overcome here (Kim et al., 2020; Zanoni et al., 2020). Considering the enormous potential as tools for introspecting human biology in health as well as in disease, ‘‘organoids’’ has been selected as ‘‘Method of the Year 2017’’ by Nature Methods^1^. Thus, organoids can also be worthy of state-of-the-art nanotoxicity evaluation. The subtle but consistent early nanotoxicity signatures at metabolite, protein, or gene expression levels can be identified by using organoid models in conjunction with several cutting-edge analytical modalities such as fluorescence-based methods, microfluidics, artificial intelligence, multi-omics integration, and single-cell analyses (Brazovskaja et al., 2019; Benning et al., 2020; Liu et al., 2020; Rodriguez-Garcia et al., 2020; Schuster et al., 2020; Tomasi et al., 2020; Costamagna et al., 2021; Duzagac et al., 2021). Thus, the utility of various organoid models to evaluate the toxicity of conventional as well as nano-drugs will be discussed under the current review along with associated challenges and future directions.

## Organoid Models and Their Applicability in Drug Toxicity Assessment

**FIGURE 3:**
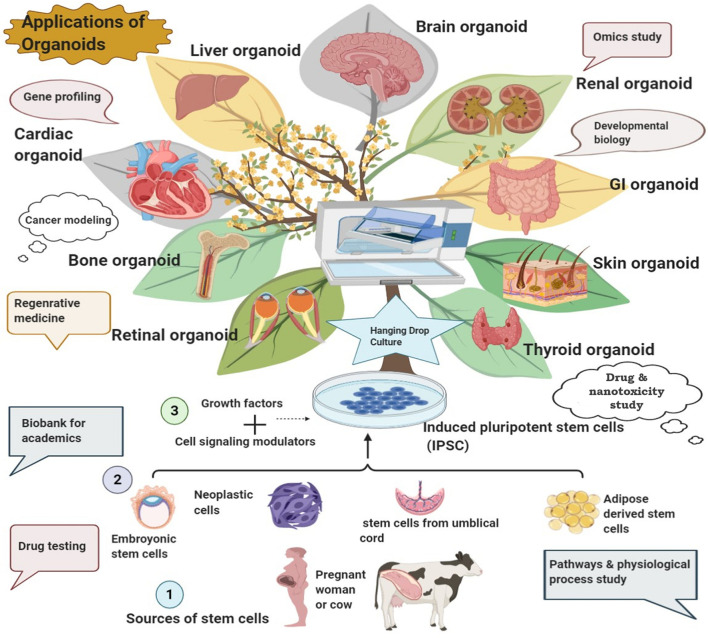
Development of various organoids from diverse sources and their potential biomedical applications.

Organoids can be customized as *in vitro* 3D tissue replica or on microchip as “collection of organ-specific cell types that develops from stem cells or organ progenitors and self-organizes through cell sorting and spatially restricted lineage commitment in a manner similar to *in vivo*” (Science 2014. 345:124). It can be developed from diverse cell origins that include embryonic stem cells (ESCs), induced pluripotent stem cells (iPSCs), adult stem cells, cancer cells, primary tissue cells, xenograft, and even mature cells (Figure 3; Bar-Ephraim et al., 2019; Syed et al., 2019; Syed and Tanwar, 2020). Decellularized extracellular matrices (ECMs) such as Matrigel, Geltrex, and Cultrex BME are usually employed for organoid development as scaffolding support mimicking the native ECM. The matrix constituents facilitate cell adhesion which can also be modulated by the inherent enzymatic machinery of the developing organoid system. These matrices have contributed immensely in various organoid developments, for instance, the development of skin organoid from human iPSCs and generation of the stem-cell-derived intestinal crypt-villus organoid, human ESC- and iPSC-derived brain organoid, gastric organoid, liver organoid, and lung organoid, all using Matrigel (Figure 3; Sato et al., 2009; Lancaster et al., 2013; McCracken et al., 2014; Miller et al., 2019; Ha et al., 2020; Lee et al., 2020). However, these matrix constituents are poorly defined with significant batch-wise variation that affects the reproducibility of organoid generation for clinical transition (Phipson et al., 2018; Aisenbrey and Murphy, 2020). Engineered organoid matrices provide an effective alternative to those conventional matrices, as they are chemically defined, tunable to specific requirements, and reproducible surmounting the limitation of batch variation to support uniform matrix-guided organoid development (Kratochvil et al., 2019; Aisenbrey and Murphy, 2020). These engineered matrices can be composed of either natural biopolymers such as collagen, alginate, hyaluronic acid, and fibrin-laminin or synthetic polymers such as poly-L-lactic acid (PLLA), polyglycolic acid (PGA), and Amikagel or recombinant elastin-like protein hydrogels (Kratochvil et al., 2019). Several organoids including the intestine, kidney, lung, liver, pancreas, and brain organoids have been developed from diverse cell origins using these engineered matrices (Cruz-Acuña and García, 2019; Kratochvil et al., 2019; Aisenbrey and Murphy, 2020; Singh and Lutolf, 2020; Hofer and Lutolf, 2021; Zhang et al., 2021). Organoids differ from spheroids in terms of the latter which is usually developed from cancer cell lines or tumor biopsies and resembles a multicellular tumor model made by non-adherent cancer cell aggregates while the former is embedded within the matrix with a more ordered configuration mimicking the respective organ (Sutherland et al., 1971; Lazzari et al., 2017; Białkowska et al., 2020; Mó et al., 2020; Velasco V. et al., 2020).

The prominent clinical applications of organoid technology include disease modeling, organ development experiments, regenerative/transplant medicine, precision medicine, and development of conventional as well as nano-drugs (Figure 3; Xu et al., 2018a). Organoids carry enormous utility in every stage of drug development experiments: from efficacy analyses, kinetic studies to toxicity assessment (Weeber et al., 2017; Takahashi, 2018; Xu et al., 2018a; Miranda and Cabral, 2020; Mó et al., 2020; Liu et al., 2021).

Organoid technology provides several advantages over conventional approaches such as the following: it not only mimics the near-physiological organ system by restoring much of the structural and functional characters of the real organ but also bears significant cellular heterogeneity, similar architecture barriers, and intercellular communication machinery providing an analogous developmental model to extend direct access for target study (Ootani et al., 2009; Takahashi, 2018; Xu et al., 2018a; Bar-Ephraim et al., 2019; Co et al., 2019; Schutgens et al., 2019; Balakrishnan et al., 2020; Kim et al., 2020; Liu et al., 2021). Further, no native immune system, lack of vasculature networks, false migratory behavior of tumor cells under variable conditions and drug influence, limited cytokine production, variation in signaling networks, and dissimilar adhesion molecule expression from the real one are certain basic limitations of cell culture-based toxicity experiments which can be addressed by the organoid model (Astashkina and Grainger, 2014). Most importantly, the species-specific difference in drug kinetics and metabolism often limits the value of animal model studies in drug development experiments which can be surmounted by the species-specific organoid models (Kratochvil et al., 2019). Apart from alleviating much of the aforementioned limitations, organoid models also extend the option for high-throughput toxicity evaluation, scaling-up, and relatively long-term adverse effect assessment of conventional as well as nano-drugs (Nagy and Robbins, 2019; Kim et al., 2020; Liu et al., 2021; Lu and Radisic, 2021). The potential role of various organoid models as a reliable modality for drug toxicity assessments has been delineated hereunder.

## Kidney Organoids

The kidney is considered a vital organ for toxicity assessment of several drugs. For instance, aminoglycoside, non-steroidal anti-inflammatory drugs (NSAIDs), contrast agents, and angiotensin-converting enzyme inhibitors (ACEIs) have been reported to produce toxic effects on the kidney (Shahrbaf and Assadi, 2015). Toxicity is inflicted through alteration in several mechanisms that include but are not limited to acute renal injury, intra-renal obstruction, interstitial nephritis, nephrotic syndrome, acid–base and electrolyte imbalance, intra-glomerular hemodynamics, and inflammatory changes in renal tubule leading to acute kidney injury (AKI), tubule-interstitial disease, and renal scarring (Shahrbaf and Assadi, 2015). Kidney organoid with nephrons, collecting duct networks, surrounding renal interstitium, and endothelial cells having different segments of nephrons have been developed by Takasato and coworkers in which the apoptotic effects of cisplatin, an established nephrotoxicant, has been assessed on different parts of the organoid (Takasato et al., 2015).

A human 3D renal organoid model developed from adult differentiated cells exhibited no significant cell death up to 14 days in the culture system along with ample expression of kidney markers like aquaporin-1 (AQP1), aquaporin-3 (AQP3), podocin, synaptopodin, and nephrin. The model has been employed for toxicity testing of certain drugs like aspirin, penicillin G, and cisplatin (Ding et al., 2020). Marked upregulation of kidney injury molecule 1 (KIM-1) and low levels of key detoxification enzyme γ-glutamyltransferase (GGT) activity have been observed only in drug-treated kidney cells and organoids along with a significant reduction in organoid viability. Dose-response curves and IC_50_ estimation have also indicated that high-dose or long-term consumption of these drugs has adverse effects on renal function (Ding et al., 2020). Thus, kidney organoids can be exploited as an efficient model for drug-induced nephrotoxicity assessment. Similarly, the expression of various emerging biomarkers such as urinary KIM-1, trefoil factor 3 (TFF-3), beta-2 microglobulin (B2mG), cystatin C (CysC), albumin (ALB), total protein (TP), clusterin (CLU), neutrophil gelatinase-associated lipocalin (NGAL), IL-18, and osteopontin (OPN) associated with different types of kidney damages can also be analyzed in this kidney organoid model for drug-induced nephrotoxicity assessment (Dieterle et al., 2010; Askenazi et al., 2011).

The human 3D organoid glomeruli model has been employed for investigating podocyte and nephronal diseases along with analysis of doxorubicin toxicity (Hale et al., 2018). The blue fluorescent protein (BFP-2)-tagged MAF bZIP transcription factor B gene (MAFB-BFP2) expression has been considered as the marker of podocyte development as a high expression of MAFB is an exclusive feature of developing podocytes. Concentration-dependent loss of BFP2 signaling, increased caspase-3 activity before cell death, and fragmentation and destruction of glomeruli along with the reduction in glomerular size have been observed as the effects of doxorubicin toxicity (Hale et al., 2018).

In another study, a marked upregulation of cytochrome P450 enzyme and Kim-1 was observed in response to acetone and cisplatin (at the clinical dose of 20 mg/kg), respectively, in kidney proximal tubule (PT) organoid (Astashkina et al., 2012). Several metabolic detoxification conjugates have been detected as cisplatin adducts such as cisplatin-GSH, cisplatin-cysteine, cisplatin-N-acetylcysteine (NAC), and cisplatin-cysteinyl-glycine (Cys-Gly). Cytokine profiling for toxicity-associated inflammatory response has depicted significant enhancement of IL-6 and MCP-1 levels in cisplatin, doxorubicin, 4-aminophenol (PAP), and colchicine exposure. Elevation in RANTES and MIP-1α has been observed against all the drugs barring PAP while IL-1β was upregulated in cisplatin and doxorubicin exposure only (Astashkina et al., 2012). This model extends the opportunity for high-throughput screening of drug-induced nephrotoxicity, kidney-related biomarker discovery, analyses of metabolic alterations, and immunological studies.

## Gastro-Intestinal Organoids

The gastrointestinal tract (GIT) crypt organoid derived from genetically modified mice has been used to analyze the drug metabolism and toxicological effect of anticancer drug camptothecin (CPT)-11 (Lu et al., 2017). CPT-11 undergoes UGT1A1-dependent glucuronidation for detoxification. Organoids derived from Ugt1 deletion mice have depicted severe susceptibility to CPT-11-induced intestinal toxicity in comparison to the control. Drug-treated organoids had shown differential xenobiotic nuclear receptor (XNR) expression for xenobiotic clearance (Lu et al., 2017). Long-lived gastric organoids analogous to the mature pyloric epithelium have been developed from single self-renewing Lgr5^+^ stem cells that sustained up to 9 months (Barker et al., 2010). The gastric organoid with gland-domain buds has been documented to secrete gastric intrinsic factor, pepsinogen C, and mucin that holds potential for modeling GI-related diseases and drug analyses (Barker et al., 2010). Similarly, intestinal organoids have been developed from human iPSCs which possess several intestinal cell types such as intestinal stem cells, enterocytes, goblet cells, Paneth cells, enteroendocrine cells, smooth muscle cells, and fibroblasts along with microvilli and tight junctions (Mithal et al., 2020; Yoshida et al., 2020; Zhang et al., 2020). The expression of different transporters like ABCB1/MDR1 has also been detected along with effective efflux transport through them. Further, the presence of the inducible CYP3A4 enzyme system justifies the potential of this organoid model in pharmacokinetic and drug toxicity assessment (Onozato et al., 2018). Similarly, human colon organoids and gastric organoids derived from the human PSCs can provide a valuable platform for modeling various enteric diseases such as colitis and colon cancer, inflammatory bowel disease (IBD), peptic ulcer, and gastric cancer (GC) due to *Helicobacter pylori* infection, viral infections, etc., along with screening and toxicological analyses of the relevant drugs (McCracken et al., 2014; Múnera et al., 2017; Lanik et al., 2018). Rectal organoids developed from the rectal epithelia of cystic fibrosis (CF) mutant subjects have been employed to observe cystic fibrosis transmembrane regulator (CFTR) functions and the response of CFTR-modulating drugs: CFTR potentiator VX-770 (ivacaftor/KALYDECO) and the CFTR corrector VX-809 (lumacaftor) (Dekkers et al., 2016). The experiment on the organoid model revealed significant dependence on the genetic and mutational factors over CFTR residual function and drug response. The drugs have depicted promising therapeutic effects in most of the mutant types in a dose-dependent manner without producing any significant organoid toxicity (Dekkers et al., 2016). Flavopiridol (100 μM), loperamide (100 μM), paracetamol (100 μM), ketoprofen (300 μM), and alosetron (100 μM) were studied to find their toxic effect on human iPSC-derived colon organoids (Su et al., 2020). The cell viability was compromised in the first two compounds, but the remaining three produced no adverse effect on the organoid cells (Su et al., 2020). Colonic organoids were developed from iPSCs derived from patients with familial adenomatous polyposis (FAP-iPSCs) along with specific germline mutation. Efficacy analyses of XAV939 and rapamycin revealed that both the compounds reduced proliferation in mutant FAP colonic organoids but the proliferation in wild-type organoids was also affected, restricting their clinical application (Crespo et al., 2017). However, aminoglycoside antibiotic Geneticin selectively targeted the mutant organoid to restore the usual proliferation, thus advocating its therapeutic application (Crespo et al., 2017). Intestinal organoids developed from the small intestinal crypts of mice were further exploited for evaluation of the cytotoxic potential of irinotecan, 5-fluorouracil, flavopiridol, and loperamide through CellTiter-Glo^®^ 3D cell viability assay following 24–72 h of drug treatment. The IC50 values of all the drugs have been calculated with significant precision, and dose-dependent cell death has been observed for all the drugs. Thus, the model can be efficiently used for drug toxicity assessment (Brandon et al., 2017).

## Pancreatic Organoids

Pancreatic organoids and spheroids have also contributed immensely for disease modeling, drug efficacy, and toxicity analysis (Bresciani et al., 2019; Gupta et al., 2020). For instance, pancreatic organoids developed from the 3D culture of a subpopulation of progenitor cells expressing high aldehyde dehydrogenase activity (ALDH^high^) and pancreatic progenitor markers such as PDX1, carboxypeptidase A1, pancreas-associated transcription factor 1a, and MYC has been transplanted in immune-deficient mice (Loomans et al., 2018). Insulin production from the transplant organoid has been detected by immunostaining along with the expression of several functional endocrine markers such as PDX1, islet amyloid polypeptide, NKX6.1, and synaptophysin. Thus, the endocrine functionality of the organoid very much resembles the actual organ, thus extending the opportunity to use this organoid in regenerative medicine and toxicological analyses (Loomans et al., 2018). The human PSC-derived acinar/ductal pancreatic organoid has been transplanted in immune-deficient mice and used as a model to study cystic fibrosis (Hohwieler et al., 2016). Several CFTR correctors and potentiators have been applied to the model to monitor restoration of CFTR function, suggesting the utility of this model in drug effect and toxicity analysis (Hohwieler et al., 2016; Wills and Drenth, 2016).

## Lung Organoids

Lung organoids have been developed from different cell types like human PSCs and alveolar epithelial progenitor cells. The source cells are differentiated properly to generate various lung-specific cell types such as myofibroblasts, goblet cells, basal cells, functional alveolar epithelial with alveolar type 1 (AT1) and AT2 cells, and lung microvascular endothelial cells (Nikolić and Rawlins, 2017; Kong et al., 2021; Vazquez-Armendariz and Herold, 2021). The organoid also depicted a lung analogous transcriptional as well as functional profile. The lung organoid has already been proved valuable in regenerative medicine and lung disease modeling along with drug efficacy as well as toxicity analyses (Dye et al., 2015; Chen et al., 2017; McCauley et al., 2017; Tan et al., 2017; Zacharias et al., 2018; Archer et al., 2021). For instance, syncytial virus tropism on the respiratory system has been explored using the lung organoid model (Collins et al., 2013; Liesman et al., 2014; Chen et al., 2017). Recently, the alveolar or lung organoid has emerged as an important model for studying host–*Mycobacterium tuberculosis* (MTB) interactions (Fonseca et al., 2017; Thacker et al., 2020). It can be a valuable alternative to animal models, as lab animals often fail to properly mimic clinical signs of tuberculosis because they are not the natural host of MTB. The lung organoid restores adequate immune functions to follow host–microbial interactions. Simultaneously, it provides the opportunity for efficacy and toxicity analyses of TB drug candidates (Li Y. et al., 2020).

Lung cancer organoids have been developed from patient tissues having different lung cancer subtypes such as squamous cell carcinoma, large cell carcinoma, adenocarcinoma, small cell carcinoma, and adenosquamous carcinoma (Kim M. et al., 2019; Hai et al., 2020; Li et al., 2020; Choi et al., 2021). Efficacy analyses of the different chemotherapeutic drugs have depicted promising responses against different cancer mutant organoids. For instance, the BRCA2-mutant type responded against olaparib, EGFR mutant to erlotinib, and EGFR-mutant/MET-upregulated organoid to crizotinib. The dose-response curve and IC_50_ analyses have depicted the significant cytotoxic effect of docetaxel (IC_50_ = 0.08 μM) over the other drugs such as olaparib (IC_50_ = 69 μM), erlotinib (IC_50_ > 100 μM), and crizotinib (IC_50_ = 3 μM). Further, docetaxel produced marked cell death in various lung cancer organoids as well as in normal bronchial organoids (Kim et al., 2019). Thus, it is evident that lung/alveolar or bronchial organoids can be efficiently used for modeling of different pulmonary diseases including several types of lung cancer along with efficacy and toxicity analyses of different chemotherapeutic agents.

## Liver Organoids

Drug-induced liver injury (DILI) is a major drawback of various drugs including but not limited to paracetamol, antituberculosis drugs, NSAIDs, penicillins and cephalosporins, sulfonamide, ketoconazole, and other azoles, as well as highly active antiretroviral therapy (HAART) (David and Hamilton, 2010). Drugs such as alatrofloxacin, alpidem, amineptine, beclobrat, bendazac, benzarone, benziodarone, flupirtine, lumiracoxib, suloctidil, and sitaxentan have been withdrawn in the past due to liver toxicity (Kocadal et al., 2019). Almost 32% of drug withdrawal took place between 1975 and 2007 due to drug-induced hepatotoxicity (Stevens and Baker, 2009). Thus, considerable attention has been directed toward the development of liver organoids as a model for analyzing hepatic disorders and preclinical evaluation of DILI (Xu et al., 2018a; Cox et al., 2020; Nuciforo and Heim, 2020; Sorrentino et al., 2020; Sun and Hui, 2020; Harrison et al., 2021; Nguyen et al., 2021). For instance, implantation of human iPSC-derived liver buds in mice generated vascularized and functional human liver organoids along with analogous gene expression and metabolite profile mimicking the human liver (Takebe et al., 2013). Metabolism of drugs like ketoprofen or debrisoquine also generated human-specific metabolites in human iPSC-liver bud transplants. This has opened the prospect of using the model for drug metabolism and toxicity analyses (Takebe et al., 2013). *In vitro* liver organoids can be generated from neonate stem cells and even from mature cells like terminally committed hepatocytes under the influence of certain molecular inducers. Such type of study carries importance in liver regenerative medicine (Katsuda et al., 2017).

An experiment by Mun et al. (2019) urges special emphasis on the current context of drug-induced hepatotoxicity assessment. Mature human hepatic organoids derived from human ESCs and induced PSCs depicted self-regeneration properties as well as strikingly similar morphological and functional attributes with the liver. Strong induction of CYP3A4 activity has been observed in the organoid as a response to drug metabolism following the treatment with rifampicin, acetaminophen, and nifedipine. Transcriptional profiling of the organoid has revealed comparable expression of phase I drug-metabolizing CYP enzymes and phase II detoxification enzymes with liver tissue (Mun et al., 2019). The organoid has also been exploited as a model to predict the toxicological effect of certain drugs and has found to be advantageous than the 2D hepatocyte culture. The 2D hepatocyte culture is unsuitable for drug toxicity assessment because the expression of enzymes related to drug metabolism rapidly disappears in this model. The cytotoxic effect of troglitazone (2 μM) and acetaminophen (1 μM) was analyzed in 2D hepatocyte culture as well as in the liver organoid model. Cytotoxic drug rotenone and/or safe compound dexamethasone were taken as reference compounds. Although the toxic effect for the two reference compounds was similar in 2D culture as well as the organoid model, the effects of troglitazone and acetaminophen differed in the two models. Cell viability as well as morphological assessment and TC50 analyses depicted that organoid is much more sensitive to troglitazone and acetaminophen in terms of toxic effect as compared to 2D hepatocyte culture. Further, the other parameters of toxic effect analyses such as ROS generation, GSH content, and nucleic structure modulation were also found to juxtapose the aforementioned results (Mun et al., 2019). The clinically relevant dose (C_max_) of the antidiabetic drug for humans is 6.29 μM which is much higher than 2 μM causing cytotoxic effects, thus underlining the basis for withdrawal of troglitazone from the market. Further, the effects of two structurally analogous antibiotics trovafloxacin (withdrawn from the market for inducing hepatotoxicity) and levofloxacin have been compared in both of the *in vitro* models. Levofloxacin produced no cytotoxic effect in either model over C_max_ of 23.8 μM while trovafloxacin markedly affected cell viability with reduced cell numbers only in the organoid model but not in the 2D culture model at 0.8 and 4 μM concentrations. Levofloxacin exerted a cytotoxic effect in the organoid model with only ≥100 μM concentration. Thus, it is evident that customized liver organoid efficiently replicates the native drug metabolism and susceptibility to drug-induced hepatotoxicity of the real liver organ. Thus, it can be used as an efficient model for drug toxicity assessment and preferred over the 2D culture model in such type of analysis (Mun et al., 2019).

Further, iPSC-derived hepatocyte organoids and 3D culture systems have also proven their potential for drug discovery, screening of small molecules, toxicity assessment, drug–host–microorganism interaction, and several other important aspects of the disease-associated clinical intervention (Ng S. S. et al., 2018; Corbett and Duncan, 2019). Vorrink et al. (2018) have screened 123 drugs for potential hepatotoxic effects with or without clinical signs of DILI in hepatic 3D spheroid cultures. The spheroids have been exposed to 1×, 5×, and 20× concentrations of C_max_ of the target compounds. None of the 53 DILI-negative compounds affected hepatocyte viability in the experimental model; however, 48 of the 70 DILI-positive compounds have been successfully identified as potential hepatotoxic candidates that significantly reduced the cell viability of the spheroid, thus yielding 69% sensitivity and 100% specificity. A step ahead, hepatocyte spheroids generated from mice, rats, and rhesus monkeys have been used for analyses of interspecies precision of the model in drug toxicity prediction. Four DILI-negative and seven DILI-positive compounds for human have been tested in the related animal models mostly revealing false results with marked interspecies variation (Vorrink et al., 2018). The experiment is a flagship, vividly elucidating the limitation of using the related animal model *in vivo* approach for drug toxicity assessment and strongly advocating the potential benefit of using the species-specific organoid/spheroid model for drug development purposes. However, spheroids have been depicted to be usually less sensitive to methotrexate, an established chronic hepatotoxin, as compared to hepatocyte monolayer cultures limiting its application in chronic *in vitro* toxicity assessment (Walker et al., 2000). The microfluidic 3D hepatocyte organ-on-chip model has facilitated on-chip IC_50_ analyses of several standard drugs such as diclofenac, acetaminophen, rifampin, quinidine, and ketoconazole. The obtained results correlated with their respective LD_50_ values, thus establishing the applicability of the model in drug toxicity assessment (Toh et al., 2009; Bhushan et al., 2013). Kostadinova et al. (2013) have also employed 3D liver coculture and 2D hepatocyte monoculture systems for the assessment of toxicity induced by several hepatotoxic drugs such as troglitazone, trovafloxacin, acetaminophen, and their respective non-toxic analogous compounds: pioglitazone, levofloxacin, and N-acetyl-meta-aminophenol. A comparison between 3D versus 2D culture systems revealed that the 3D system is more sensitive to drug-induced hepatotoxicity as compared to the 2D culture system. The 3D culture also provided much closure to *in vivo* toxic effects than the 2D culture system. Further, the drug-induced toxicity pattern was markedly different in human and rat 3D culture systems. For instance, troglitazone produced cytotoxicity to reduce the cell viability only in human 3D culture but not in the rat 3D liver culture system. This interspecies variation in drug response also urges the requirement of a species-specific drug analysis model (Kostadinova et al., 2013).

## Brain Organoids

The development of the brain organoid is another crucial achievement in the arena of organoid technology. This not only is helpful in the modeling of several neuropsychiatric, neurodegenerative brain disorders, developmental disorders, neurotropic infectious diseases, and tumors but also serves as an efficient model for toxicological assessment of several drugs, precisely those which can surmount the blood–brain barrier (Di Lullo and Kriegstein, 2017; Chuye et al., 2018; Chen et al., 2020; Shou et al., 2020; Velasco et al., 2020; Sun et al., 2021). The brain organoid model has a definitive advantage over cell culture-based studies as the organoid can act as a direct source in terms of targeted species and personalization, heterogeneity, and interaction analysis (Qian et al., 2018). Various organoids have been developed to mimic either whole-brain or sub-brain regions such as the hypothalamus, adenohypophysis, forebrain, midbrain, cerebral cortex, cerebellum, and hippocampus or even neural organoids (Suga et al., 2011; Lancaster et al., 2013; Muguruma et al., 2015; Sakaguchi et al., 2015; Jo et al., 2016; Qian et al., 2016, 2018; Quadrato et al., 2017; Zhu et al., 2017; Mansour et al., 2018; Paşca, 2018; Xu et al., 2018a; Andrews and Nowakowski, 2019; Cakir et al., 2019; Marton and Paşca, 2019; Nascimento et al., 2019; Chen et al., 2020). The organoids have also facilitated the evaluation of efficacy and toxicity of the drugs having implications over the neuromuscular system (Vaez Ghaemi et al., 2018; Marx, 2020; Matsui et al., 2020; Shou et al., 2020). The human PSC-derived cerebral organoid has been successfully used as a model to study the Zika virus-induced teratogenic effects on the developing brain and the therapeutic effects of the potential candidates which can alleviate those (Watanabe et al., 2017). Zika virus-induced apoptosis, innate immune responses including chemokine and cytokine production, inflammatory responses, and growth restriction along with neural destruction throughout the central nervous system have been observed in the organoid model. Cholesterol 25-hydroxylase (CH25H) has enhanced the protection against the virus. This is because the enzyme promotes the conversion of cholesterol to 25-hydroxycholesterol (25HC) which boosts up the natural host defense. However, subsequent increase in the 25HC concentration has only moderate effects without much reversal of cell death indicating mild toxicity of the compound with enhanced exposure. The effect of antibiotics duramycin, ivermectin, and azithromycin to combat Zika virus infection has also been analyzed. Duramycin and ivermectin depicted a strong antiviral effect. However, ivermectin slightly increased cell death which was not observed for duramycin, whereas azithromycin was unable to reduce the viral infection. Thus, besides efficacy analyses of the potential drugs, the organoid model can also be used for drug toxicity assessment (Watanabe et al., 2017). An experiment on the retinal organoid model derived from iPSCs of patients having retinitis pigmentosa (RP) due to frameshift mutation in the RPGR gene has depicted that CRISPR/Cas9-mediated genetic correction can alleviate the disease to a great extent (Deng et al., 2018). Another study employing cerebral organoids derived from human iPSCs with DISC1 mutation showing psychiatric disease has revealed concomitant WNT agonism, which can be reversed by WNT antagonism (Srikanth et al., 2018). Such type of genetic study has extended the scope to use the organoid model for drug-induced genotoxicity assessment. Further, the 3D cell culture chip of human neural progenitor cells also facilitates an alternative drug toxicity analysis module as a related state-of-the-art technique (Kafi et al., 2015; Nierode et al., 2016; Wang et al., 2018).

## Cardiac Organoids

Preclinical test for predicting side effects, including proarrhythmic and cardiotoxic effects, is another important toxicological analysis in drug discovery. This is because several drugs have been reported to exert cardiotoxicity such as astemizole, chlorphentermine, cloforex, propoxyphene, grepafloxacin, pergolide, nifedipine, naftidrofuryl, and rimonabant and eventually withdrawn from the market (Kocadal et al., 2019). Engineered human cardiomyocytes and cardiac organoids developed from diverse sources have demonstrated their worth in cardiac disease modeling, regenerative and precision medicine, and drug-induced cardiotoxicity assessment (Eder et al., 2016; Voges et al., 2017; Goldfracht et al., 2019). Disease-specific cardiomyocytes developed from human iPSCs suffering from hereditary long QT syndrome (LQT), familial hypertrophic cardiomyopathy (HCM), and familial dilated cardiomyopathy (DCM) revealed increased sensitivity to cardiotoxic drugs cisapride and nicorandil as compared to the control (Liang et al., 2013). Human iPSC-derived cardiomyocytes have been used for the evaluation of cardiotoxicity of four tyrosine kinase inhibitor drugs: crizotinib, sunitinib, nilotinib, and erlotinib. Among them, the former three established cardiotoxic drugs produced potent adverse effects on the cardiomyocytes. These drugs have reduced cell viability, enhanced apoptosis, increased ROS generation, yielded metabolic alteration, and impacted ion channel functions. In contrast, cardiac-safe erlotinib inflicted minor impact on the cardiac cells corroborating with its existing safety profile (Doherty et al., 2013). Further screening of 24 drugs on the model has depicted no structural as well as functional cardiotoxic effect for all the known cardiac-safe drugs while 16 out of the 18 drugs with known cardiac implication affected structural or functional integrity of the cardiomyocytes (Doherty et al., 2015). Similarly, human iPSC-derived cardiomyocytes, endothelial cells, and cardiac fibroblasts have been employed for toxicity evaluation of several cardiotoxic tyrosine kinase inhibitor drugs. A “cardiac safety index” has been developed based upon their cardiotoxic effects to facilitate a high-throughput screening modality for the potential candidates (Sharma et al., 2017). The cardiotoxic effect of mitomycin C inhibiting the proliferation of diverse cell types in the *in vitro* developed cardiomyocytes or cardiac organoids has also been observed (Voges et al., 2017). Cell viability, apoptosis, injury marker LDH, troponin I contractile force analysis, electrical stimulation activity, impedance, T2 relaxation time, ROS generation, calcium handling and signaling, metabolic alteration and activation of death signaling, fibrosis, and hypertrophy, are the usual attributes which have been monitored to evaluate the extent of drug-induced cardiotoxicity on diverse cardiac organoid models (Doherty et al., 2013, 2015; Sharma et al., 2017). For instance, toxic response of drugs 4-aminopyridine, erythromycin, bepridil, desipramine, and quinidine on rat-engineered cardiac tissues has generated T2 prolongation, after-contractions, and arrhythmia. However, the poor sensitivity of rat-engineered cardiac tissues to cardiotoxic drugs often raised doubts regarding the authenticity of the toxicity model (Eder et al., 2014). Similarly, the toxicological effects of pharmacological agents isoproterenol acting as β-adrenergic agonist and E-4031 as hERG blocker has been analyzed on the human IPSC-derived cardiac microphysiological system along with the clinically established multi-ion channel blocker drug verapamil and a β-adrenergic antagonist metoprolol. Gene expression, morphological study, and electrophysiological measurements revealed good coherence and similarity with the clinical toxicological implications induced by the candidates, as observed in the earlier introspection (Mathur et al., 2015). The potential of the heart organoid model has further been reinforced as human cardiac organoid efficiently working to elucidate hypoxia-enhanced doxorubicin cardiotoxicity (Richards et al., 2020).

## Skin Organoids

Skin is the largest as well as the most superficial organ to endure diverse physical and chemical assaults, particularly from topical formulations. Skin organoids generated from diverse sources bear paramount importance in several skin-related disease modeling, regenerative medicine, and drug efficacy as well as toxicity testing (Lei et al., 2017; Lee et al., 2020; Lee and Koehler, 2021). The developmental process of the mouse skin organoid deciphers valuable insight in regenerative medicine as it prospers the path of microenvironmental reprogramming toward restoration of the self-organizing property of adult skin. Supplementation of diverse molecular modulators such as PKC inhibitors, growth factors like IGF2, IGFBP3, or VEGF2 and signaling pathway regulators like Wnts and MMPs in a timely manner regulate the transition of the dissociated cells from newborn mouse skin to the hair-bearing skin (Lei et al., 2017). In contrast, adult cells as a source succumb to achieve complete development and stalls only forming cellular aggregates. However, the outlook toward drug toxicity analyses in this model urges further investigation (Lei et al., 2017). Similarly, skin organoid containing hair follicles, sebaceous glands, and adipocytes has also been developed from mouse pluripotent stem cells (Lee J. et al., 2018). Induction of hair follicle formation and growth has been depicted to be induced by treatment with TGFβ inhibitor SB431542, recombinant BMP4, FGF-2, and BMP inhibitor LDN-193189 (Lee et al., 2018). The human skin organoid containing hair follicles and glands has also been customized from human PSCs by modulation of FGF and TGFβ signaling pathways. Grafting of the organoid in nude mice yielded planar hair-bearing skin formation (Lee et al., 2020). These organoids can also be employed as an excellent model to elucidate the process of pigmentation, hair folliculogenesis, and induction of hair growth along with exploring the mechanisms of inhibitory drugs and their potential toxic effects. For instance, a human skin equivalents-on-a-chip platform has been employed for the evaluation of barrier function and doxorubicin toxicity on skin in a handy as well as resource-effective manner (Abaci et al., 2015). The skin-on-a-chip modality has also been effectively exploited for testing of several drugs like dexamenthasone, penicillin, salicylic acid, caffeine, and isosorbide dinitrate along with several cosmetics containing UV-ray protecting nano-formulations. It provides an efficient platform for evaluating drug absorbance, epidermal drug delivery, efficacy, and toxicity (Lee et al., 2017; Mori et al., 2017; Rodriguez-Garcia et al., 2020). Inflammation of the skin equivalent induced by TNF-α enhanced the expression of proinflammatory cytokines such as IL-1β, IL-6, and IL-8 affecting the integrity of the skin tight-junction structure that can be protected by the anti-inflammatory drug dexamethasone (Wufuer et al., 2016). Exposure of the cosmetic chemicals sodium lauryl sulfate and steartrimonium chloride with the microfluidic skin equivalent inflicted toxic effects by hindering the angiogenesis, reducing keratinocyte proliferation, and inducing apoptosis of the keratinocytes as the signatures of barrier disruption and altered cell viability (Jusoh et al., 2019). Another similar instance has depicted the utility of the *in vitro* skin model to elucidate the action of penicillin and neutrophil migration under *Staphylococcus aureus* infection (Kim et al., 2019). Similarly, the toxic effect of hair dye containing azo group compound Basic Red 51 (BR51) showed cytotoxic effects and generation of ROS on human keratinocytes (Zanoni et al., 2014). In another experiment, a human 3D-skin-melanoma spheroid model has been employed for evaluating cytotoxic effects of anticancer drugs and also provides a comparative analysis regarding the therapeutic efficacy of the treatment regimen in 2D culture as well as in the 3D spheroid model. In the treatment of cancer cells with tumor necrosis factor-related apoptosis-inducing ligand (TRAIL) in combination with either sublethal scale ultraviolet-B exposure or cisplatin, both the regimens elicit cytotoxic response to the cancer cells in 2D culture; however, 3D spheroid cells are significantly killed only by TRAIL/cisplatin combination and no significant cytotoxicity was inflicted by the TRAIL/ultraviolet-B module (Vörsmann et al., 2013). This introspection vividly signifies the supremacy of using the 3D organoid model over the 2D culture system for drug efficacy and toxicity analysis.

## Other Organoids

Prostate organoids developed from human ESCs depicted similar architecture and functional attributes of the human prostate gland. The model has also shown that low-dose exposure of bisphenol A perturbed the prostate morphogenesis which can be replicated *in utero* for the potential developmental anomaly (Calderon-Gierszal and Prins, 2015).

Primary human testicular cells are also found to self-organize to develop human testicular organoids containing spermatogonia, Leydig cells, Sertoli cells, peritubular myoid cells, and germ cells along with tight-junction protein expression. Testosterone and inhibin B have been detected in the model along with the secretion of several cytokines. The model has the potential to be exploited for the treatment of infertility as well as in drug efficacy and toxicity assessment (Baert et al., 2017; Sakib et al., 2019a, b). Similarly, 3D human testicular organoids have been used for the assessment of reproductive toxicity induced by anti-mitotic compounds such as busulfan, cisplatin, doxorubicin, and etoposide. A dose-dependent reduction in cell viability has been observed for all the compounds with enhanced Caspase 3/7-mediated apoptosis. The 3D organoid has depicted a higher IC_50_ value than the corresponding 2D culture for all the candidates, thus acting as an efficient alternative for testicular drug toxicity evaluation (Pendergraft et al., 2017). The human testicular organoid has also been successfully used as a model for Zika virus pathogenesis. Therapeutic effects of antiviral drugs against the viral infection and their reproductive toxicity potential can also be investigated using this organoid model (Strange et al., 2018). The testicular 3D organoid exposed to male reproductive toxicant mono(2-ethylhexyl) phthalate experienced a dose-dependent increase in germ cell autophagy. The germ cells in the 3D organoid perceived less stress than in 2D culture, thus further establishing the utility of the organoid in drug-induced reproductive toxicity assessment (Sakib et al., 2019b).

Customization of blood vessel organoids from human PSCs containing important morphological and physiological attributes like vascular smooth muscle cells and pericytes including similar gene expression profile of the native blood vessels is another noteworthy development which can facilitate better nutrient and gas exchange to other organoids for increased survival in an engineered multi-organoid integrated platform (Wimmer et al., 2019a; Markou et al., 2020; Mori et al., 2020). Blood vessel organoids have found immediate attention as a potential *in vitro* model for introspecting vasculature-associated diseases and toxicity analyses of different systemic drug candidates. For instance, the blood vessel organoid has been employed to model diabetic vasculopathy and to monitor γ-secretase inhibitor N-[N-(3,5-difluorophenacetyl-L-alanyl)]-S-phenylglycine t-butyl ester (DAPT)-mediated inhibition of expansion and thickening of the basement membrane of blood vessels. The model has also enabled toxicity assessment of the drugs to facilitate drug discovery for various rare genetic vascular diseases, atherosclerosis, and cancer (Wimmer et al., 2019a). The blood vessel organoid has also facilitated elucidation of novel therapeutic checkpoints in terms of identifying DLL4 and NOTCH3 as crucial potentiators of diabetic vasculopathy (Wimmer et al., 2019b). Further, the vasculogenic and angiogenic potential of several growth factors and the antagonistic action of their inhibitors can also be evaluated using such luminal organoid system which carries significant therapeutic interest (Virumbrales-Muñoz et al., 2020). A microfluidic chip-based atherosclerosis model has successfully identified the cytotoxic effects of the anti-atherosclerotic drug probucol which has gone overlooked in the petri-dish culture, thus justifying the supremacy of such *in vitro* 3D-organoid model over the 2D culture modality (Zheng et al., 2016).

The spectrum of retinal diseases is quite diverse with devastating consequences, which urged the development of retinal organoid to serve as an efficient model for evaluating various eye-related disorders as well as preclinical efficacy and toxicity analyses of diverse ophthalmic drug candidates (Achberger et al., 2019b; Brooks et al., 2019; Chichagova et al., 2019; Kim et al., 2019; Gao et al., 2020; Kruczek and Swaroop, 2020; Lukovic et al., 2020). For instance, the retinopathic adverse effects of the anti-malaria drug chloroquine and the antibiotic gentamicin have been elucidated on a human iPSC-derived retina-on-a-chip model in a dose-dependent manner (Achberger et al., 2019b). Application of the drug 4-hydroxytamoxifen and moxifloxacin produced photoreceptor degeneration while curcumin provided protection against oxidative stress in a retinal organoid model similar to the observations of *in vivo* experimentation (Chang et al., 2014; Ito et al., 2017; Hallam et al., 2018). Further, drug efficacy analyses employing the retinoblastoma organoid model have depicted that topotecan alone or in combination with melphalan effectively contains mitotic proliferation of the tumor cells while methotrexate was almost ineffective to restrict tumor growth (Saengwimol et al., 2018). The tetinal organoid can also extend an efficient and reliable *in vitro* model for testing the ocular toxicity of several systemic therapeutics like anti-TB drugs rifabutin, rifampin, and ethambutol, anti-malarial drug hydroxychloroquine, and immunosuppressants tacrolimus and cyclosporine to facilitate novel drug discovery (Achberger et al., 2019a).

The engineered palatal fusion HWJSC/HPEKp organoid model has been used to evaluate the effect of different cleft palate teratogens (Belair et al., 2018). Theophylline, triamcinolone, and valproic acid showed significant disruption in organoid fusion, while tributyltin chloride and all-trans retinoic acid have inflicted cytotoxicity to fusing organoids. Compounds K02288 (BMP inhibitor) and BMS536924 (IGF inhibitor) served as the positive control, also inhibiting the fusion. Significant inhibition in epithelial migration of cells at 24 h post-treatment was observed in erlotinib-, K02288-, and BMS536924-treated organoids whereas reduction in cell viability was observed in erlotinib, CH5183284, and RO4929097 treatment (Belair et al., 2018). Thus, the model can be useful for teratogenic disease modeling along with the screening of corrective drugs and their potential toxic effects.

Magnetic 3D bioprinting (M3DB) technology is used to customize secretory epithelial organoids from the human dental pulp stem cell (hDPSC). The salivary gland organoid system depicted precise structural architectures, intracellular ATP activity, and inducible α-amylase activity. The organoid has been advocated to be valuable in regenerative medicine and treating the case of radiotherapy-induced xerostomia (Adine et al., 2018).

A human primary cell- and stem cell-derived multi-organoid “body-on-a-chip” system containing several organoids such as liver, cardiac, lung, vascular, testis, colon, and brain has been developed to serve as a screening platform of several drugs with or without toxicity potential (Skardal et al., 2020). Cell viability, cytotoxicity, ATP activity, and heartbeat assays were considered for monitoring the toxic effects of the compounds. FDA-recalled drugs bromfenac, tienilic acid, and troglitazone produced hepatotoxicity; astemizole, cisapride, mibefradil, and terodiline produced significant cardiotoxicity while pergolide, rofecoxib, and valdecoxib depicted mild toxicity, but an increase in dose enhanced the adverse effects. Further, 48-h exposure of loratadine and quercetin produced cell death in the liver and cardiac organoid only at higher than recommended plasma concentrations in human whereas aspirin induced some amount of cell death only in cardiac organoid at an excess of the clinical dose. Exposure to the drugs at clinically relevant doses revealed a non-toxic outcome. Thus, this integrated organoid platform can serve as an efficient model for preclinical drug toxicity assessment (Skardal et al., 2020).

## Cancer Organoids

The cancer organoid model can be helpful for initial screening of the carcinogenic/tumorigenic potential of the candidate drugs to ensure drug safety (Kocadal et al., 2019). Organoid technology can be exploited for two-way purposes. Firstly, normal organoids can be used for evaluating the expression of cancer-specific markers in the organoid cells after treatment with drug candidates. Secondly, cancer organoids can be used as a model to study the chemotherapeutic potential of various anticancer drugs along with an assessment of their toxic effects on the organoid cells (Evans, 2015; Chatzinikolaidou, 2016; Mittler et al., 2017; Boucherit et al., 2020; Gupta et al., 2020).

Pancreatic ductal adenocarcinoma patient-derived pancreatic organoids PDO 163 and PDO 185 have been employed for therapeutic efficacy analysis of anticancer drugs gemcitabine, irinotecan, and paclitaxel. PDO 163 depicted the response to gemcitabine and irinotecan treatment while it was non-responsive to paclitaxel therapy unlike PDO 185 which showed a broader response to all three chemotherapeutic agents. Efficacy analyses of all three drugs in the respective *in vivo* mouse models yielded a similar therapeutic response, as predicted in the *in vitro* organoid model (Baker et al., 2016; Frappart et al., 2020).

Patient-derived organoids from colorectal and gastroesophageal cancer patients have been used for screening of various drugs either in different phases of clinical trials or in clinical practice to evaluate their chemotherapeutic effects and toxicity. Lapatinib was the most effective against ERBB2-amplified organoid while the AKT1-amplified E17K mutant organoid was the sole among the bunch which strongly responded to AKT inhibitors. The BRAF V600E mutant organoid depicted decreased viability following treatment with BRAF inhibitor vemurafenib. Overall, the drug response observed in various organoid models followed the clinical therapeutic response history of the respective type of cancer patients, thus elucidating the robustness of the model for drug testing (Vlachogiannis et al., 2018). The tumor organoid model developed from low- and high-grade appendiceal cancer patients has also been employed for screening the therapeutic response of 5-fluorouracil, oxaliplatin, FOLFOX, FOLFIRI, or regorafenib. The models hold potential for predicting the therapeutic outcome of certain treatment regimens along with their potential toxic side effects against precise cancer types (Votanopoulos et al., 2018).

Therefore, it is evident from the aforementioned instances that organoid technology is an excellent alternative to conventional methods for implementation in disease modeling, regenerative and personalized medicine, drug screening, and toxicity testing. Although as a nascent tool organoids are so far mostly being used for toxicity assessment of traditional drugs and almost remain virgin for evaluation of the adverse effects of nano-drugs, considering the benefits and potential of the organoid model in this arena, it is not too far to be used as a common platform for nanotoxicity assessment.

## Nanotoxicity Assessment Using the Organoid Model

The global NP drug market is predicted to achieve a valuation over US$ 200 billion by 2024 at a CAGR of 10% according to the 2018 analysis of “Research And Markets” while as per the 2017 prediction of Grand View Research, Inc., the anticipated valuation of the global nanomedicine market will reach US$ 350.8 billion by 2025 at a CAGR of 11.2% (Grand View Research, Inc., 2017; Research And Markets, 2018). This enormous prospect of nanotechnology in the biomedical and pharmaceutical industry is self-explanatory to realize the extent of nano-exposure to the global population looming ahead (Yan et al., 2020; Ji et al., 2021). Investigation of short- as well as long-term nanotoxicity has gained considerable attention in this context to ensure the nano-safety aspect. Diverse conventional toxicity assessment platforms, particularly the 2D culture model, have been extensively used to elucidate the landscape of toxicity imparted by diverse nanomaterials along with their underlying mechanisms (Wani et al., 2011; Azhdarzadeh et al., 2015; Armstead and Li, 2016; Fadeel, 2019; Abdelhamid, 2020; Shinde et al., 2020). However, minute alteration in the physicochemical properties of NPs such as shape, size, charge, crystal structure, surface area, surface functionality, and customization protocol can distinctly change the toxicity profile of any established nano-formulation (Zhu et al., 2012; Jeevanandam et al., 2018). Further, dose, duration and route of exposure, and even the type of cell, organ, or organism receiving the NP exposure are also crucial determinants of the extent and type of nanotoxicity (Park et al., 2010; Lankoff et al., 2012; Berger et al., 2013; Wang et al., 2013; Gliga et al., 2014; Chen et al., 2015; Schmid and Stoeger, 2016; Budama-Kilinc et al., 2018; Saini and Srivastava, 2018). As nanotoxicity is a multifactorial event, therefore, the choice of the evaluation method or model is crucial for proper qualitative or quantitative evaluation. For instance, the toxicity assessment of a potential cardiotoxic nano-drug on the HepG2 cell line will furnish false indications. Similarly, a nano-drug tested in the rat model may not properly predict its toxic potential for humans because of the interspecies variation in drug metabolism (Toutain et al., 2010; Liu et al., 2017). Further, the sensitivity to toxicity varies considerably between 2D cell culture and 3D organ models (Chia et al., 2014). As 2D cell cultures are having a relatively short life, they can thus only predict the short-time nanotoxic effects (Kapałczyńska et al., 2016). In contrast, 3D organ models have a relatively longer survival, so nanotoxic effects for even a month of exposure can be elucidated by employing the modality. Moreover, considering the bulk of nano-drugs in the pipeline and enhanced nanointervention under circumstances requiring rapid drug or vaccine development like the ongoing COVID-19 pandemic, the rapid preclinical nanotoxicity screening method is urgently required (Al-Halifa et al., 2019; Chauhan et al., 2020; Erasmus et al., 2020; Pang et al., 2020; Shin et al., 2020). The subject-specific 3D organoid model mimicking the key vital organs of the target organism or an integrated multi-organoid model can serve as a fast as well as reliable preclinical nanotoxicity screening method with several advantages over the conventional modalities (Table 2). The organoid technology has already started to contribute to nanotoxicity assessment, and the key developments in this arena have been delineated hereunder.

**TABLE 2:** Potential organoid models for toxicity assessment of different nanoparticles.

Potential toxic NPs	Model used for toxicity analyses	Possible response(s)	Model organoid
Methoxy poly(ethylene glycol)-polycaprolactone (MePEG-PCL)	Human liver carcinoma cells (HepG2)	Size-dependent internalization	Liver organoid
ZnO-NPs	Human hepatocyte (L02)	DNA damage, cell membrane disruption, decrease cell viability, oxidative stress, mitochondrial damage	
Ti-NPs + Ag-NPs	Male Wistar rats	Reduce mitochondrial activity	
Poly (lactic-co-glycolic acid) polyethylene glycol nanoparticles (PLGA-PEG NPs)	Human liver carcinoma cells (HepG2)	Lysosome disruption causing DNA damage and cell death	
Ag-NPs, ZnO-NPs	Human liver carcinoma cells (HepG2)	DNA damage	
Ag-NPs	Primary liver cells of mice Male CD-1 (ICR) mice Sprague Dawley rats	Affects cell viability Hepatocyte necrosis, hepatobiliary toxicity Inflammatory reactions in liver Induces hepatocellular damage	
Cu-NPs	Male SD rats	Liver injury due to inflammation and oxidative stress	
PVP-Ag NPs	Male C57BL/6 mice, Male SD rats	Liver damage due to inflammation and inhibitory fatty acid oxidation	
TiO_2_-NPs	Male albino mice, SD rats, Male mats rats, C57/BL6 mice, ICR mice	Oxidative stress, inflammation, DNA damage, potential apoptotic mechanisms, cellular infiltration, hepatocyte necrosis, etc.	
NiO-NPs	Male Wistar rats	Activation of NF-κβ signaling pathway, Oxidative stress	
Silica	HepG2	Increase ROS Mitochondrial damage & oxidative stress	
Iron oxide	Human hepatocellular carcinoma cells	Reduced cell viability	
Cadmium telluride quantum dots	HepG2,	Cell viability reduction	
Cadmium selenide quantum dots coated with MAA, BSA/EDAC, and EGF	Rat primary hepatocytes	Cell death	
Liposomes	HepG2	Cytotoxicity *via* lipid metabolism	
Carbon (C_60_)-NPs	HepG2	Cytotoxicity, leaky cell membrane	
Ag-NPs	Buffalo rat liver cells (BRL-3A)	Decrease cell viability Increase LDH and ROS	
Dendrimers	BRL-3A, HepG2, H4IIE (rat hepatoma)	Cytotoxicity	
Polyacrylic acid (PAA) coated Iron oxide NPs	CD1 mice	Inflammatory reaction, induces oxidative stress	
ZnO-NPs	Human embryonic kidney (HEK293) cells	Mitochondrial dysfunction, reduction of SOD, depletion of GSH, and oxidative DNA damage	Kidney organoid
Fullerenes	HEK293	DNA damage	
Multi-walled carbon nano tube (MWCNTs) with pristine	HEK293	Upregulation of cell apoptosis proteins	
Single-walled carbon nano tubes (SWCNTs) coated with pristine	HEK293	Arrest of cell cycle	
Dendrimers	COS-7 (African green monkey kidney)	Cytotoxicity	
Polyester-based nanocarriers	A498 (human kidney carcinoma)	Cytotoxicity	
CuO NPs	HEK293	Altered ROS, reduced cell viability	
QD	HEK293	Apoptosis	
CuO NPs	A 459	Oxidative stress, Genotoxicity	Lung organoid
MWCNTs with carbonyl (CdO), carboxyl (COOH), hydroxyl (OH)	Human lung-tumor cell lines (H596, H446, and Calu-1)	Altered cell viability	
MWCNTs with pristine	Human embryonic lung fibroblasts (IMR-90)	Cytotoxicity	
SWCNTs SWCNTs with pristine	Human alveolar carcinoma epithelial cell line (HACEC); Normal human bronchial epithelial cell line (NHBEC), Human alveolar epithelial cells, A549	Cell death Cytotoxicity, activation of NF-κB signaling pathway	
Smaller CNTs	Fetal lung tissue	Cytotoxicity	
Ag-NPs	Human alveolar cell line	Reduced cell viability, increase ROS	
	Sprague–Dawley rats	Inflammatory and cytotoxic effects in lungs	
	Brown Norway and Sprague–Dawley rats	Acute pulmonary neutrophilic inflammation with the production of proinflammatory and pro-neutrophilic cytokines, compromised lung function	
	Human lung epithelial cells (BEAS-2B)	Genotoxicity	
Silica-NPs	Human bronchoalveolar carcinoma cells	Increase reactive oxygen species (ROS), increase LDH, Increase Malondialdehyde	
Zinc oxide-NPs	Human bronchial epithelial cells	Reduce cell viability, increase oxidative stress	
Titanium oxide-NPs (TiO_2_)	Human lung cells	Oxidative stress, DNA adduct formation, cytotoxicity	
Cerium oxide(CeO_2_) NPs	Human lung epithelial cells (BEAS-2B)	Cell death, increase ROS and oxidative stress	
SPION	Human lung epithelial cells (A549)	Stimulation of JNK, activation of tumor necrosis factor-alpha (TNFα), decrease in NF-kβ, production of ROS	
QD	Human lung adenocarcinoma cells	Mitochondria-dependent cellular apoptosis, decrease cell viability	
Liposomes	Male Han Wistar rats	DNA damage, genotoxicity	
Cationic liposomes	A549 cell line	Reduced cell viability	
Copper(II)-conjugated phosphorus dendrimers Chitosan nanoparticle PLGA-NPs	MCR5 (proliferative human lung fibroblasts)A549 lung epithelial cells Lung bronchial epithelial cells (BEAS-2B cells)	Cytotoxicity Cell necrosis	
Aluminum oxide NPs	Human brain microvascular endothelial cells (HBMVEC)	Mitochondrial dysfunction Oxidative stress and cell death	Brain organoid
Cadmium telluride quantum dots	Human neuroblastoma cell	Cell viability reduction	
Quantum dots (QD)	Neuron like PC12 cells	Cell death and axonal degeneration	
Carbon (C_60_)-NPs	Neuronal human astrocyte	Cytotoxicity, leaky cell membrane	
Carbon nanotubes	PC12 cells	Induces ROS, decreases mitochondrial membrane potential and superoxide dismutase (SOD)	
Dendrimers	N2a (mouse neuroblastoma cells), mHippoE-18 (mouse embryonic hippocampal cells), primary neural cell cultures, intracerebroventricular injection in mice	Cytotoxicity, decrease cell viability, apoptosis in brain cells	
Copper(II)-conjugated phosphorus dendrimers	U87 (human glioblastoma–astrocytoma, epithelial-like)	Cytotoxicity	
Ag-NPs	rBMEC (primary rat brain microvessel endothelial cells)	Pro-inflammatory cytokine release, increases permeability and cytotoxicity of cells	
Fe_2_O_3_-NPs	Growing neuron cell line PC12	Decrease growth	
CdSe-NPs	Primary rat hippocampal neuron cells in culture	Decrease of cells viability	
Superparamagnetic iron-oxide nanoparticles (SPION)	Murine neural stem cells	DNA damage, dissipated cell-membrane potential, hyperpolarization of the mitochondrial membrane, altered activities of SOD	
TiO_2_, ZnO, Fe_2_O_3_, Al_2_O_3_, CrO_3_	Neuro-2A	Apoptosis	
TiO_2_-NPs	Wistar albino rats, female ICR mice	Genotoxicity due to DNA damage, increase creatine kinase, increased levels of troponin T, altered heart parameters	Cardiac organoid
ZnO NPs	CD-ICR mice, Sprague–Dawley rats, Wistar albino rat	DNA damage, myocardial degeneration, necrosis focal fibrosis in heart tissue, fatty degeneration in cardiovascular cells, reduction in heart rate, etc.	
Ag-NPs	Wistar rats, Sprague–Dawley rats, Albino zebrafish, Oryzias latipes (medaka) embryos	Oxidative stress, increased superoxide anion production in heart tissue, myocardial ischemia, significant decrease in heart beats, pericardial edema, separation of myofibrils, cardiac oxidative stress etc.	
	BALB/C mice	Oxidative stress, DNA damage, apoptosis in heart	
	Sahul India Catla catla heart cell line (SICH)	Induce oxidative stress, cytotoxicity, and genotoxicity	
SWCNTs	Rats, C57BL/6 mice	Myofiber degeneration, heart tissue damage, aortic mtDNA damage, inflammatory responses, and oxidative stress	
MWCNTs	Male C57BL/6J mice	Myocardial infarction	
Silica NPs	Sprague–Dawley rats, zebrafish, Wistar rats	Myocardial ischemia, altered cardiac rhythm, increased cardiac troponin-T, pericardial edema, inflammatory reaction, oxidative stress, and ROS generation	
TiO_2_NPs, ultrafine titanium dioxide (UFTiO(2)	Rats	Cardiac structural damage leading to heart failure Increases cardiac protein phosphorylation	
Iron oxide NPs	Human cardiac microvascular endothelial cells (HCMECs)	Decrease in cell viability	
Ultra-small superparamagnetic iron oxide nanoparticles	Mice	Oxidative stress, generation of reactive oxygen species and superoxide dismutase in heart	
Zinc oxide	Human colon carcinoma cells	Altered oxidative stress, reduce cell viability, expression of inflammatory biomarkers	Intestinal organoid
Dendrimers (PAMAM)	Caco-2 (colon adenocarcinoma cells), SW480 (primary adenocarcinoma cells of colon)	Cytotoxicity	
Copper(II)-conjugated phosphorus dendrimers	HCT116 (human colon cancer)	Cytotoxicity	
Ag-NPs	Mice	Damages epithelial cells of microvilli as well as intestinal glands	
	SW480 cells Caco-2 cells	Cell death Mitochondria toxicity in intestinal epithelial cells	
TiO_2_-NPs	SW480 cells	Cell death	
ZnO-NPs	Human intestinal Caco-2 and SW480 cells	Cytotoxic, Cell death	
CuO NPs	Rat small intestine epithelial cells (IEC-6)	Cytotoxicity due to formation of ROS that damages mitochondrial membrane	
Liposomal 5-fluoro-2′-deoxyuridine (FUdR)-dipalmitate	Mice	Loss of columnar epithelial cells and enlarged nuclei with prominent nucleoli in these cells, granulocyte infiltration, and presence of cell debris in ileum	

A murine kidney organoid derived from a 3D proximal tubule culture has been employed to explore the nephrotoxicity induced by hydroxylated generation-5 PAMAM dendrimer (G5-OH) and gold NPs of less than 6-nm size (Astashkina et al., 2012, 2014). A dose-dependent response with limited cytotoxicity was evidenced after 48 h of G5-OH dendrimer exposure, resulting in a maximum of 20% cell death at the highest tested concentration (0.8 mg/ml). However, no significant upregulation of lysosomal enzyme N-acetyl-β-D-glucosaminidase (NAG) was observed indicating meager or little toxicity of the G5-OH dendrimer on the 3D proximal tubule organoid. Analyses of Kim-1 and TNF-α as an indication of proximal tubular epithelial cell damage revealed that the G5-OH dendrimer mediated significant induction at 0.675 mg/ml dose as compared to the untreated culture. Known nephrotoxic compound cisplatin (1.7 mM) was taken as the positive control. As an indication of the inflammatory response, estimation of different cytokines IL-1b, IL-2, IL-6, IL-10, TNF-α, INF γ, MCP-1, MIP-1α, MIP-1β, MIP-2, and RANTES from a 48-h G5-OH dendrimer-exposed 3D proximal tubule culture at 0.675-mg/ml concentration revealed no significant upregulation in most cases between G5-OH dendrimer and untreated culture except IL-2, IL-10, and MIP-1α. However, a significant variation in the cytokine level was observed between cisplatin and G5-OH dendrimer-treated culture for most of the parameters. This indicates that the G5-OH dendrimer induces some sort of nephrotoxicity, although the extent is very little as compared to the clinically evidenced nephrotoxic drugs (Astashkina et al., 2014). Gold NPs extensively adhered to the HA gel matrix of the 3D organoid culture, thus limiting its transport and biodistribution within the culture media to elicit any toxic effect. Further, the experiment also compared the 3D organoid results with the *in vivo* animal model experiment. Mice were injected with the G5-OH dendrimer, and cell viability was assayed in the proximal tubular culture developed from the harvested kidney of the injected mice. The cell viability was little influenced by the treatment and almost behaved like the untreated control. The results are comparable with the *in vivo* model experiment where kidney accumulation of the G5-OH dendrimer postinjection has been observed affecting BUN or creatinine clearance. Thus, it is evident that the 3D proximal tubule organoid model efficiently imitates the nanotoxicity effects obtained from the *in vivo* experiment (Astashkina et al., 2014). Further, a comparative analysis between 3D kidney organoid culture and 2D kidney cell cultures has been performed for their ability to mimic the *in vivo* drug-induced nanotoxicity effects. Considerable similarities in terms of inflammatory cytokine production, Kim-1 expression, modulation of cytochrome enzymes, TNF-α production, and NAG shedding have been observed between 3D kidney organoid culture and *in vivo* model in response to cisplatin which strikingly differs in 2D kidney cell cultures (Astashkina et al., 2012). The aforementioned discussion confers that the 3D kidney organoid model can yield a much similar nanotoxicity prediction to the *in vivo* system as compared to the 2D cell culture model.

The hepatotoxic effect of 50-nm carboxylated polystyrene particles has been assessed by using a “GI tract–liver–other tissues” body-on-a-chip device or a multi-tissue microphysiological system. The system comprised enterocytes (Caco-2) and mucin-producing cells (TH29-MTX) as representative of the human intestinal epithelium and HepG2/C3A cells to mimic the liver. Various concentrations ranging from 15 to 480 × 10^11^ NPs ml^–1^ of media with 24-h exposure have been set for inducing nanotoxicity. Estimation of toxicity-induced cytosolic enzyme release depicted increased aspartate aminotransferase (AST) in all three cell types; however, HepG2/C3A cells released more AST as compared to the other two cell types, whereas low release of alanine aminotransferase (ALT) and GGT was observed only in HepG2/C3A cells. Despite significantly increased AST release in HepG2/C3A cell culture due to NP-induced hepatocellular injury, fluorescent live/dead staining was unable to detect it in terms of cell viability alteration. Moreover, the experiment also suggested that sensitivity of the hepatic cells to NP toxicity increased when operated along with the intestinal epithelial cells, depicting the importance of an integrated multi-organoid system to attain more precision in nanotoxicity evaluation (Esch et al., 2014; Choi et al., 2016).

The silver NPs (AgNPs) sized between 40 and 130 modulated liver metabolic functions like cytochrome P450-mediated detoxification and urea production in decellularized MSC spheroid derived from HepG2 culture. NPs with larger diameters were less toxic than the smaller ones. However, aggregation of NPs interferes with their biodistribution and entry into the cells (Bidon et al., 2017).

Hepatocyte-based organoid or 3D monoculture systems have been predominantly used for the assessment of nanomaterial-induced hepatotoxicity; however, the importance of non-parenchymal cells is often overlooked. In this context, the decisive role of Kupffer cells in driving overall hepatotoxic response against nanomaterial exposure and in turn the advantages of using 3D hepatocyte coculture microtissue (MT) model over 3D hepatocyte monoculture models have been vividly highlighted by Kermanizadeh and associates (Kermanizadeh et al., 2019b, 2020). Kupffer cells are mostly localized at the sinusoidal lumen, thus exclusively interacting with the gut antigens, and the payload was delivered through the portal vein. Evidently, these cells crucially regulate hepatic immune response as well as the toxicity profile induced by NPs delivered through diverse routes. The coculture MT models depicted a higher overall nanotoxicity profile as compared to monoculture models in terms of cytotoxicity, caspase 3/7 activity, and pro/anti-inflammatory cytokine response upon exposure with different concentrations of ZnO, Ag, multi-walled carbon nanotubes, and TiO_2_ nanomaterials for variable durations. Further, two coculture MT models derived from two different donors used for the study also depicted significant variation in nanotoxicity profile suggesting individual variation in toxicity response (Kermanizadeh et al., 2019b). Most importantly, the utility of the 3D hepatocyte coculture MT model for assessing long-term toxicity from prolonged exposure of bio-sustainable nanomaterials has been highlighted by the research group which is physiologically more pragmatic than evaluating nanotoxicity induced by single exposure of NPs at a higher dose (Kermanizadeh et al., 2019a, b, 2020). The 3D human liver MT spheroid model was found to be suitable for nanotoxicity estimation arising from low-dose repeated exposure of ZnO, TiO_2_, and CeO_2_ nanomaterials. The nanomaterials inflicted very little cell death even after 10 repeated exposure for up to 3 weeks, but considerable aging effects arise afterward, rendering the model unfit beyond the period. Despite the well-known importance of AST as a hepatic injury marker, the enzyme failed to indicate subtle hepatotoxicity induced by the nanomaterials. Further, estimation of pro/anti-inflammatory cytokines (IL6, IL8, IL10, and TNF-α) depicted the limitation of such *in vitro* 3D liver MT model as it failed to mimic the *in vivo* regenerative potential of the liver during the given 1 or 2 weeks of recovery period (Kermanizadeh et al., 2019a). Probably, as there was no stemness left among the cells of the differentiated 3D microtissues, they cannot regenerate after nanotoxic effects imprinted. Effective methods to restore the regenerative potential of the *in vitro* liver organoid model are essential to overcoming such limitation. Supplementation of liver progenitor cells may be an alternative approach in this direction which needs to be explored further (So et al., 2020).

A gastric cancer patient-derived organoid model has been employed to explore the antitumor effect of established anticancer nano-drug albumin-bound paclitaxel NP or nab-paclitaxel. This is a commonly used injectable therapeutics in breast cancer, lung cancer, and pancreatic cancer, etc. The IC_50_ of nab-paclitaxel was lowest as compared to 5-Fu and epirubicin in all three GC-organoid models. An increase in exposure time up to 48 h augmented apoptosis in all three types of GC organoids (Xiao et al., 2020).

In another instance, the mouse ISC-derived intestinal organoid has been used as a carrier of 5-aminosalicylic acid-loaded PLGA NPs for targeted therapy of IBD. Incubation of the organoid with the NPs did not affect the organoid growth and cell viability even after 7 days (Davoudi et al., 2018). Similarly, DNA-functionalized AuNPs encapsulated into mouse ISC-derived intestinal crypts organoid has been employed for targeted delivery of the nano-drug to carry out gene regulation therapy of IBD. Incubation of the intestinal organoid with the nano-drug for even up to 7 days produced little cytotoxic effect (Peng et al., 2015).

A tumor-on-a-chip system composed of spheroids integrated with a microfluidic system supporting the normal physiological flow was used to evaluate NP transport in the tissue (Albanese et al., 2013). This is because aggregation of nanomaterials with the 3D culture matrix components often limits their biodistribution and intracellular penetration, restricting nanotoxicity evaluation using the 3D organoid or spheroid model (Albanese et al., 2013; Astashkina et al., 2014). PEGylated AuNPs having a variable hydrodynamic diameter of 40, 70, 110, or 150 nm were investigated for their penetration in the spheroid tissue. An increase in diameter hinders tissue penetration of the NPs, as it is a diffusion-dependent process. Functionalization of the NPs with transferrin converts the passive process to a receptor-targeted active process increasing the tissue uptake of the ligand-bound NPs (Albanese et al., 2013). This platform can also be valuable for the toxicity assessment of the nano-drugs in the preclinical screening process.

The blood–brain barrier (BBB) is impermeable to most of the conventional drugs whereas nano-drugs can penetrate through it, treating CNS and brain disorders (Sokolova et al., 2020; Kumarasamy and Sosnik, 2021). However, NP-induced neurotoxicity needs to be evaluated properly using a suitable toxicity evaluation model. The *in vivo* animal model differs considerably from humans in anatomical and physiological perspectives whereas 2D cultures are inefficient to replicate the complex networking, thus often failing to reflect the real event (Martignoni et al., 2006; Jensen and Teng, 2020). The 3D human brain organoid or spheroid model offers an efficient alternative for nanotoxicity evaluation of CNS targeting nano-drugs and disease modeling (Wang, 2018; Klein et al., 2020; Shou et al., 2020). Human iPSC-derived brain spheroids and 3D LUHMES model mimicking human dopaminergic neurons have been used to evaluate the toxic effects of sodium citrate or polyethylene glycol (PEG)-functionalized AuNPs and polymeric polylactic acid (PLA) NPs at various concentrations with a 24–72-h exposure. All the NPs were internalized in both the 3D models, but the internalization of AuNPs in 3D LUHMES was delayed. Mitochondrial membrane potential analyses depicted an acute response for the NPs in brain spheroids whereas 3D LUHMES model sensitivity enhanced with an increase in concentration and exposure time. NP exposure enhanced the expression of several oxidative stress-related genes in brain spheroids, with Au-PEG eliciting maximum response whereas 3D LUHMES was less responsive. Au-PEG produced the strongest effect with significant downregulation of all the tested cytokines in 3D LUHMES cultures while it was indifferent to the other two NPs. However, brain spheroids did not respond as stoutly to Au-PEG depicting downregulation in some cytokines and chemokines. Thus, AuNPs and PLA NPs both can induce toxicity in a concentration-dependent manner and the brain spheroid model has been depicted as more suitable for general neurotoxicity assessment as it contains glia providing neuronal support (Leite et al., 2019).

A microfluidic chip-based early-stage atherosclerosis model mimicking the attributes of blood vessels has been employed to evaluate the anti-atherosclerosis effect of platinum-NPs (Pt-NPs) alongside an *in vivo* mouse model for comparative analyses. The Pt-NPs demonstrated excellent activity to contain ROS production induced by the hyperglycemic condition alleviating atherosclerosis. The anti-atherosclerotic activity of Pt-NPs on the chip-based model juxtaposed with the observations in the animal model study justifying the reliability of 3D-organoid models in drug discovery and toxicity analysis (Zheng et al., 2016).

Free gemcitabine, multifunctional squalenoylated gemcitabine NPs (SqGem-CholPEG NPs), and abraxane (nab-paclitaxel), all three chemotherapeutic agents for pancreatic cancer, have been tested for their cytotoxic effect in monolayer-cultured KPC cells or monolayer-cultured KPC cells plated in Matrigel in 3D or 3D KPC organoid culture in Matrigel at different concentrations for a 48-h exposure (Tucci et al., 2018). The cell viability assay indicated a dose-dependent response for the first two drugs except for abraxane which showed similar toxic effects at all the experimental doses. Overall, the organoid and 3D culture depicted less sensitivity to all the drugs as compared to monolayer culture. This may have resulted from inappropriate drug penetration or variation in drug sensitivity of the diverse cell types in the organoid (Tucci et al., 2018).

### Biomarkers for Nanotoxicity Evaluation in Organoid Models

The multi-arrayed mechanisms of nanotoxicity induce alterations in morphological attributes, cell viability, functional parameters, gene expression profile, proteomic and metabolomic profile, signaling pathways, etc (Gioria et al., 2016; Kim et al., 2017; Carrow et al., 2018; Zheng et al., 2018; Hufnagel et al., 2020). Nanotoxicity varies considerably depending upon the employed organoid model, age of the organoid or spheroid, and type of NP as well as its dose, route, and duration of exposure (Figure 1; Henriksen-Lacey et al., 2016; Wu et al., 2017; Eilenberger et al., 2019; Poli et al., 2020). The type of toxicity signature emanates from the organoid cells vividly correlated with the molecular mechanisms of the nanotoxicity. The selection of assay should be intended to identify those toxicity signatures as early as possible post-exposure. For instance, nephrotoxicity inflicted by the G5-OH PAMAM dendrimer on the kidney organoid has been evaluated through cell viability assay to identify DNA and membrane integrity, immunohistochemistry, or immunoassay to detect nephrotoxicity-associated biomarkers like Kim-1 and CYP2E1, expression analyses of cytokines like MCP-1, TNFα, INFγ, RANTES, MIP-1α, IL-6, IL-10, and IL-1β to evaluate inflammatory response, and estimation of NAG shedding to detect necrosis of proximal epithelial cells (Astashkina et al., 2012, 2014). Flow cytometric analysis was performed for PAX8, LHX1, SIX2, SALL1, or WT1 which serve as human kidney differentiation markers to ensure proper development of the human kidney organoid to be employed for toxicity assessment (Morizane et al., 2015). Blood urea nitrogen (BUN), EPO, GGT production, etc., can be evaluated for the analysis of nanotoxicity-induced altered kidney function. Similarly, morphological analysis using electron microscopy, cell viability analysis by MTT assay, caspase assay, release of hepatic enzymes such as AST, ALT, LDH, and GGT, aberrant cytochrome P450-mediated detoxification, albumin synthesis, and upregulated proinflammatory cytokine synthesis can be enumerated as markers of NP-induced hepatotoxicity (Lee et al., 2009; Esch et al., 2014; Choi et al., 2016). Generation of ROS enhanced malondialdehyde (MDA) production as an indicator of lipid peroxidation, diminished the superoxide dismutase (SOD) level, and elevated the level of pro-inflammatory cytokines such as IL-1α, IL-6, and IL-8 which can be analyzed as the markers of nanotoxicity in skin organoids (Chen et al., 2019). Altered production of insulin, amylase, lipase enzymes, ALDH activity, expression analysis of endocrine functional indicators PDX1, NKX6.1, and synaptophysin, etc. can serve as potential markers for nanotoxicity evaluation in pancreatic organoid (Sasaki et al., 2020). Analysis of cell viability, apoptosis, injury marker LDH, cardiomyositis indicators such as cardiac troponin T, α-actinin, and CD45/CD14/CD31, ROS generation, altered contractile or ion channel function, and calcium signaling can be carried out as potential biomarkers for NP-induced cardiotoxicity (Doherty et al., 2013, 2015; Sharma et al., 2017). Cell viability assay, apoptotic assay by Annexin V-FITC/PI staining, CCK8 assay, cytochrome P450 gene expression, analysis of xenobiotic metabolism, and detoxification-related genes can serve as the potential toxicity markers in GI organoids (Lu et al., 2017). Similarly, analysis of cell viability, morphology, mitochondrial membrane potential, expression of proinflammatory cytokines, chemokine and growth factors, and ROS generation can be carried out for the evaluation of nanotoxicity in the brain organoid model while expression of brain-associated developmental and functional markers like CORIN, OTX2, MAP2, and TBR can be analyzed to authenticate proper customization and functionality of the brain organoid (Leite et al., 2019). The potential biomarkers as well as the assay methods for the assessment of nanotoxicity in different organoid models are shown in Figure 2 and Table 3. Although the parameters for nanotoxicity evaluation seem to be diverse and according to the employed organoid model, however, analysis of cell viability, apoptosis, morphological evaluation, expression of oxidative stress-related genes, ROS production, estimation of pro-inflammatory cytokines, and organ-specific enzyme analysis can be considered as general methods for toxicity assessment of diverse nano-formulations in suitable organoid models.

**TABLE 3:** Biomarkers and assays for nanotoxicity evaluation in 3D organoid models.

Organoid	Markers	Indication	Analysis
Liver	Cell viability, morphology, Albumin, Bile, Cytochrome 450, ALT, GGT and AST, Catalase and carbamoyl phosphate synthetase-1 isoforms, and proinflammatory cytokines	Cytotoxicity, liver secretion, xenobiotic clearance, and oxidative stress	MTT assay, electron microscopy, biochemical, and gene expression study
Kidney	Apoptosis, morphology, Blood urea nitrogen (BUN), EPO, γ-glutamyltransferase production, SIX2, NPHS1, SAL1, WT1, PAX2, Bmp4, CITED1, KIM-1, trefoil factor 3 (TFF-3), beta-2 microglobulin, cystatin C, albumin, total protein, clusterin, neutrophil gelatinase-associated lipocalin, IL-18 and osteopontin, proinflammatory cytokines, and ROS generation	Cell viability, Kidney function, secretion, development and regeneration test, and oxidative stress	MTT assay, electron microscopy, biochemical, ELISA, and gene expression study
Heart	Alpha-actin, CD45, CD31, LDH, troponin, and heart beat assay	Cardiomyositis, contractile and ion channel function	Biochemical, ELISA, gene expression study, and ECG
Brain	Proinflammatory cytokines, ROS generation, CORIN, OTX2, MAP2, and TBR	Oxidative stress, Brain developmental analysis	Gene expression study, immunoassays
GIT	CyP3A4,CYP450, *Cyp2b10*, *Cyp3a11*, ABCB1/MDR1, and XPGs including *alcohol dehydrogenase Adh1*, *aldehyde dehydrogenase Aldh1a1*, and *Aldh1a7*, secrete gastric intrinsic factor, pepsinogen C, and mucin	GIT function test for detoxification, xenobiotic clearance and enzyme secretion	Gene expression study and ELISA or biochemical assay
Lung	KL6, MMP1&7, PFT ABG, and DLCO	Lung function and structural integrity analysis	Biochemical, ELISA, and gene expression study
Pancreas	Insulin, amylase, lipase, LDH, PDX1, NKX6.1, Synaptophysin, and islet amyloid polypeptide	Pancreas endocrine function, enzyme assay, and developmental assessment	Biochemical, ELISA and gene expression study
Tumor	M2-PK, Wnt Signaling pathways molecules, Her2, cytology, copy number alterations (CNAs), hematoxylin and eosin (H&E) staining, live dead cell, ATP measurement, and Annexin V-FITC/PI staining	Tumor-specific test	Gene expression study, Western blotting, immunohistochemistry, and microscopy
Testis	Testosterone and inhibin B	Function test	ELISA, Western blotting, and immunohistochemistry
Dental epithelial organoids	α-Amylase activity	Function test	ELISA, Western blotting, and immunohistochemistry

### Comparison Between 2D Monolayer Culture and 3D Organoid Culture Models for Nanotoxicity Evaluation

Toxicity and inflammatory responses induced by 25-nm zinc oxide NPs (ZnO NPs) have been investigated in 3D colon cell spheroids derived from a SW480 human colorectal cancer cell line and an NCM460 normal human colon mucosal epithelial cell line. Further, the extent of nanotoxicity has been compared in 2D and 3D cell culture systems (Chia et al., 2014). The 3D culture of NCM460 cells depicted less sensitivity to ZnO NPs with a small rise in cytoplasmic ROS level as compared to the 2D culture of NCM460. However, the 2D and 3D cell cultures of the SW480 cancerous cell line behaved differently from the normal cell line due to already existing high basal levels of intracellular ROS without any significant further rise in response to the NPs. Strong upregulation of inflammatory cytokines IL-1β and IL-18 was observed in the 2D culture of NCM460 cells as compared to the 3D culture of the NCM460 cells depicting increased susceptibility of the 2D culture of normal cells to nanotoxicity than the 3D culture system. However, NP-induced cytokine response in the SW480 cancerous cell line depicted an opposite pattern to the normal cell line with more expression of inflammatory cytokines in 3D culture than the 2D culture system. This is mostly due to the cancerous microenvironment and precise activation of downstream signaling pathways. The genotoxic effect of ZnO NPs has been evaluated through analyses of histone phosphorylation resulting from DNA damage. 2D culture of both the cell lines and 3D culture of the SW480 cancerous cell line depicted significant DNA damage whereas the 3D culture of the NCM460 normal cell line documented considerable resistance to NP-induced DNA damage. The ZnO NPs also significantly affected the morphology and viability of the cells in 2D culture whereas the 3D culture system depicted more resistance. Thus, overall findings indicated that the extent of nanotoxicity is dependent on the cell types and microenvironment. In general, 2D monolayer culture usually overestimates the extent of nanotoxicity whereas the 3D spheroid/organoid model provides more closure and realistic *in vivo*-like estimation (Chia et al., 2014).

In cancer photothermal therapy, the effectiveness of AuNPs as gold-graphene hybrid nanomaterial (Au@GO) loaded with doxorubicin was introspected in HeLa/HUVEC cell-derived 3D multicellular tumor spheroids showing more selectivity toward the fast-dividing HeLa cells as compared to the 2D culture model (Lee et al., 2018).

A 3D spheroid culture model produced from mesenchymal stem cells of human adipose tissue (hAD-MSCs) has been employed for the toxicity evaluation of CdTe/CdS/ZnS quantum dots (Qdots) (Ulusoy et al., 2016). Morphological investigations revealed a significant dose-dependent effect of the Qdots on spheroid cells with a concomitant increase in cell death. Exposure time was also found to be a crucial determinant. ATP assay for cell viability depicted a concentration-dependent effect in both monolayer 2D cell culture and 3D spheroid; however, the former showed a biphasic response which may be due to the presence of two different cell types with varying sensitivity while the latter produced a monophasic response. IC50 has depicted that the monolayer 2D cell culture is more sensitive to CdTe/CdS/ZnS Qdot toxicity as compared to 3D spheroid (Ulusoy et al., 2016). However, several previous reports have depicted that no toxicity is induced by CdSe/ZnS core/shell Qdots or carboxylated graphene Qdots in a rat model after several days of treatment, which contradict the results obtained from 2D cell culture and 3D spheroids (Hauck et al., 2010; Nurunnabi et al., 2013). This wide gap between the imperfect animal model and insufficient 2D monolayer culture model in toxicity testing can be bridged by using a target-species-specific 3D organoid or spheroid model to obtain a more realistic pre-clinical evaluation.

EpiKutis^®^ is a 3D epidermal model which has been employed for toxicity assessment of AgNPs. Toxicity was inflicted in a dose-dependent manner affecting cell viability by enhanced LDH release, exerted oxidative stress through elevated ROS generation, higher MDA production, diminished SOD level, and generation of pro-inflammatory cytokines such as IL-1α, IL-6, and IL-8. Further, a comparative evaluation of nanotoxicity between the 3D epidermal model and 2D keratinocyte culture elucidated that the former depicted more realistic analyses with improved barrier function and lesser NP penetration than the latter one at the same dose of AgNP exposure (Chen et al., 2019). Similarly, the EpiDerm^TM^ skin organoid model has been employed for genotoxicity assessment of 16- and 85-nm BASF Levasil^®^ silica NPs along with providing a comparative analysis with 2D monolayer culture. Significant reduction in cell viability along with pronounced genotoxic effects was imparted to the cells of 2D monolayer culture in a dose-dependent manner while the parameters remained almost unaffected in the 3D organoid model under similar exposure doses. Lesser penetration of NPs due to improved barrier function of the collagen layers in the EpiDerm^TM^ skin organoid model may be the underlying reason (Wills et al., 2016). Thus, designing of toxicity analysis experiment based upon an *in vitro* 2D cell culture model may often provide erroneous toxicity prediction leading to false drug screening outcome.

Comparative toxicity analyses of CdTe and AuNPs in 3D liver spheroid and 2D culture model has elucidated that the 3D model is less susceptible to the NP-induced cytotoxicity as compared to the 2D culture due to its more tissue-like morphological and phenotypic attributes (Lee et al., 2009).

Unlike conventional drugs, measurement of permeation of the nano-drugs is beyond the scope of a simple 2D cell culture model whereas 3D organoid culture can measure it (Hosoya et al., 2012; Van Zundert et al., 2020). This is very important for efficacious drug dose measurement; otherwise, it may lead to not only suboptimal drug supply but also toxicity. The *in vitro* 3D culture model of tumor fibrotic tissue from pancreatic cancer/normal fibroblast has elucidated that increase in cultured cell layers and NP size will reduce the permeation of the nano-drugs (Hosoya et al., 2012).

Poor drug penetration and inconsistent biodistribution can also result from non-specific patchy deposition of nano-drugs at the off-target tissue surfaces before the target organ leading to deposition toxicity of NPs which often pose a limitation for nanotoxicity evaluation in the animal model study (Hougaard et al., 2010; Chakraborty et al., 2016). High surface reactivity of the NPs renders them susceptible for deposition at the tissue surface of the delivery sites. Consequently, inhaled TiO_2_ nano-formulations tend to deposit at the lung airways of the mice while Ag-BSA NPs showed deposition propensity at the zebrafish skin (Hougaard et al., 2010; Chakraborty et al., 2016). Localized deposition of the NPs induces ROS production and inflammatory response leading to deposition toxicity (Brohi et al., 2017). The organoid model can overcome such limitations of the animal model study as organoid mimicking the target organ is directly exposed to the nanoformulation, leaving little opportunity for off-target drug deposition. Further, modulating the fluid flow to the organoid by a microfluidic device or 3D-bioprinted luminal channel can also assist to enhance the bioavailability of the nano-drug to the organoid (Ao et al., 2020; Chen et al., 2020). This can also alleviate the deposition toxicity issue. A comparative analysis regarding the attributes of different nanotoxicity assessment models has been depicted in Table 4.

**TABLE 4:** Comparative analysis regarding the attributes of different nanotoxicity evaluation modalities.

Attributes	2D monolayer culture model	3D organoid model	Lab animal model
Ethical issues	No ethical restriction	Lucid ethical restriction, only limited ethical issues arise pertaining to stem cell research and stem cell therapies	Stringent adherence to the ethical guidelines for animal experimentation is compulsory
Economics of operation and maintains	Least	Moderate to high depending upon the experimental requirements	Resource intensive
Batch variation in replicates	Least batch variation under predefined experimental set up	Low to moderate batch variation depending upon the matrix material and customization protocol	Moderate to high individual variation based upon the pathophysiological and nutritional status of the animals
Survival	Survival in days thus unsuitable for long-term toxicity analysis	Moderate lifespan, usually up to few months which can be enhanced by vascularization	Enough lifespan, even suitable as chronic toxicity assessment model
Efficiency to mimic the real *in vivo* condition of the target species	Very limited as it is devoid of spatial architecture, immune system, and communication machinery, etc.	Considerably efficient to mimic the near-physiological microenvironment, possess several structural and functional attributes of the real target organ, most importantly organoid from the target species or patient-derived organoid can be used to nullify interspecies variations in drug metabolism, even suitable for developing personalized medicine	Provides real *in vivo* condition but substantial interspecies anatomical and metabolic variations, particularly in drug metabolism, diversity in omics attributes often yields false prediction in the targeted species
Feasibility for structural and functional integrity study	Least	Optimum	Comprehensive
Scope for drug penetration and biodistribution analysis	Very limited to none	Multilayered organoids provide ample opportunity for drug penetration and biodistribution analysis	Most suitable model for such requirement
Cellular heterogeneity	Minimal to negligible	Considerable cellular heterogeneity is present	Extensive
Level of cell-to-cell interaction	Minimal	Optimum	Comprehensive
Tissue-native immune system interaction	None	Optimum	Extensive
Scope for organ–microenvironment interaction	None	Optimum and can be regulated depending upon the requirement	Comprehensive and regulated
Feasibility for organ–organ interaction	None	Not possible for organoid recapitulating a single-type organ, but possible in multi-organoid-on-a-chip microfluidic platform or co-customized multiple organoid system connected by vasculature/luminal organoid	Extensive
Cell-blood vessel interaction	None	Only in vascularized organoid	Yes
Fluid flow perfusion	No	Only in vascularized organoids or organoid-on-a-chip microfluidic platform or organoids connected by 3D-bioprinted lumens	Yes
Deposition toxicity issue of nano-drugs	No deposition toxicity issue of nanoparticles arise	Minimum possibility of deposition toxicity for testing the nano-drugs	Frequent probability of deposition toxicity for testing the nano-drugs
Patient-specific model/personalized medicine	Partial using specific cell lines	Most appropriate *in vitro* model	Very limited to none

## Challenges and Future Prospect

Evaluation of nanotoxicology in the organoid model tends to converge two nascent promising techniques: nanotechnology and organoid technology. Thus, the limitations of the two techniques also congregate at this interface rendering the task relatively difficult. However, considering the benefits and technological advancements that took place in these areas in recent times, it does not seem implausible but a tricky one. The bottlenecks can be summarized as follows (Truskey, 2018; Xu et al., 2018a, b). First, customization of organoids in a reproducible manner demands extreme precision in standardization even in the presence of engineered matrices, while it is almost impossible using poorly defined decellularized extracellular matrices. Achieving the reproducibility level fitful for clinical bio-translation of the organoids requires the mandatory following of good manufacturing practices like the other pharmaceutical products, stringent specification of the ingredient as well as critical quality attributes of the customized organoids, and strict adherence to the predefined bioprocessing methods, customization protocols, and ethical guidelines approved by any international regulatory authorities (Kim et al., 2020; Tran et al., 2020). Even the methods for scalability should comply with the international standard. Spheroidal age is also an important determinant of drug response and toxicity which needs to be specified to obtain a reproducible outcome (Eilenberger et al., 2019). Secondly, organoids are generally deficient in several structural and functional components of the respective organ and are thus only capable of partly mimicking their *in vivo* counterpart (Truskey, 2018; Xu et al., 2018a). Reduction in the off-target cell population which may differentiate into non-specific cell types and dominate the target cells in aged organoid culture is necessary. This can be achieved through a standardized directional differentiation protocol which requires comprehensive prior knowledge about the biochemical milieu of the interacting microenvironment as well as the cross-talking machinery. Further integration of the multi-organoid system in a single platform connected by blood vessels like luminal organoids can facilitate improved communication and better functioning of the customized organoid (Kim et al., 2020). Third, complete differentiation and full maturation of the organoid under the *in vitro* system often remain beyond the scope (Truskey, 2018; Xu et al., 2018a, b). Inadequate supply of nutrients, growth factors, and bioactive molecules, poor gaseous exchange, and stagnation of the wastes often limit the differentiation and maturation process (Tran et al., 2020). Customization of organoids along with vasculatures using microfluidics platform or 3D bioprinting method or co-customization with blood-vessel organoid or fused organoid provides the promising means to overcome the limitation (Ao et al., 2020; Chen et al., 2020; Zhang et al., 2020; Daly et al., 2021). Fourth, most of the organoids sustain for a limited time, which needs to be increased for at least one or a few months to support the analyses of long-term nanotoxicity (Xu et al., 2018b). Growing organoids in the presence of a vascular network can also assist to extend the survival of the organoid. Fifthly, nanomaterials often aggregate with the ECM components limiting intracellular penetration and biodistribution of the nano-drugs (Albanese et al., 2013; Astashkina et al., 2014). Use of a predefined testing module with specific media components which allow uniform distribution and adequate penetration of the nano-drug should be followed to avoid such complication. Sixth, the *in vitro* organoid systems usually lack physiological flow which is essential for nano-drug distribution and penetration. Prevascularized organoid constructs with a connected luminal network on microfluidic platforms or organoid-on-a-chip modules can restore the physiological flow to facilitate drug penetration and subsequent biodistribution (Kim et al., 2020). Seventh, the bioactive components of matrix materials and media may alter metabolic and gene expression and signaling pathways of the organoid system (Tran et al., 2020). Again, stringent regulatory control over the organoid customization components and protocols can solve the issue to a significant extent. The list of challenges may be further lengthened, but the point that needs special mention is an urgent requirement of developing the prevascularized organoid model employing precisely standardized globally accepted protocol. Every physicochemical property of the nano-formulation should be predefined or classified as well as the characteristics of the organoid model. Proper guidelines should be there mentioning the most suitable organoid type for a certain class of nano-drugs to evaluate their potential toxic effects. A persistent effort has been extended to resolve the issues as reflected by a handful of literature on the current context, mostly explored within the last 5–6 years and several more expected shortly.

Nanotechnology has progressively matured to contribute significantly in almost every aspect of clinical medicine with quite a few nano-formulations having been already approved by FDA and several ones which are in the pipeline (Flühmann et al., 2018; Patra et al., 2018; Prasad et al., 2018; Abed et al., 2019). Even the recent COVID-19 pandemic has been tried to battle out with nano-vaccine-based interventions, as it facilitates either nano-adjuvant-based potentiated vaccines or nano-carrier-mediated vaccine delivery (Editorial Nature, 2020). Nanomaterial-based slow-release implant devices have also augmented the vaccine efficacy by controlled sustained release of the immunogenic candidates (Shin et al., 2020). The nano-path has also been followed for even diagnosis and therapy of the SARS-CoV-2 infection with considerable success (Chauhan et al., 2020; Dormont et al., 2020; Lammers et al., 2020; Moitra et al., 2020; Udugama et al., 2020). The steep increase in nano-interventions certainly demands a rapid and reliable nanotoxicity assessment module to maintain the ongoing flurry of nano-drugs in the market. The organoid technology has enormous potential with several proven examples to perfectly fit in the facet of urgency as an efficient preclinical model for *in vitro* nanotoxicity evaluation with considerable *in vivo* essence.

## Conclusion

The emergence of organoid technology and its establishment as a standard model for drug screening and toxicity assessment will certainly help to fast-track nanotechnology-based vaccine and diagnostic and therapeutic developments by sorting out several age-old limitations of conventional methods. The scope of organoid technology even spans beyond drug discovery with a significant potential contribution to disease modeling and regenerative and personalized medicine. Thus, convergence of the two state-of-the-art technology evolving nano-interventions in the organoid model will certainly produce novel breakthroughs in the biomedicinal arena.

## Author Contributions

MP conceptualized, critically analyzed, and improved the manuscript. MG, RK, LB, and AK wrote the manuscript. All authors contributed to the article and approved the submitted version.

## Conflict of Interest

The authors declare that the research was conducted in the absence of any commercial or financial relationships that could be construed as a potential conflict of interest.

## Publisher’s Note

All claims expressed in this article are solely those of the authors and do not necessarily represent those of their affiliated organizations, or those of the publisher, the editors and the reviewers. Any product that may be evaluated in this article, or claim that may be made by its manufacturer, is not guaranteed or endorsed by the publisher.

## Footnotes

1
https://www.nature.com/collections/qtwnlymxzh
